# Ionomic and metabolic signatures governing sterility mosaic disease resistance in pigeonpea

**DOI:** 10.3389/fpls.2026.1870334

**Published:** 2026-09-03

**Authors:** Promil Kapoor, Mamta Sharma, Kasi Rao Mediga, Shubham Saini, Dipak Kshirsagar, Shweta Chhabra

**Affiliations:** 1 Department of Plant Pathology, Chaudhary Charan Singh Haryana Agricultural University, Hisar, India; 2 Legumes Pathology, Accelerated Crop Improvement, International Crops Research Institute for the Semi-Arid Tropics, Patancheru, India; 3 Department of Bio-Nanotechnology, Chaudhary Charan Singh Haryana Agricultural University, Hisar, India

**Keywords:** biochemical stress markers, FTIR spectroscopy, ICP-MS, pigeonpea, PPSMV-2, resistance breeding, sterility mosaic disease

## Abstract

**Introduction:**

Sterility mosaic disease (SMD) represents a critical biotic constraint that severely limits pigeonpea yields throughout South Asia. The disease is incited by *Pigeonpea sterility mosaic emaravirus* (PPSMV), which is actively transmitted by the eriophyid mite vector *Aceria cajani*.

**Methods:**

Host-pathogen interactions were comprehensively analyzed using a multi-disciplinary approach. This included field screening, molecular and microscopic diagnostics along with biochemical, spectroscopic and elemental profiling.

**Results:**

Over two growing seasons, genotypes ICPL 20096-1 and ICPL 99090 exhibited high resistance, while the susceptible check (ICP 8863) exceeded 90% disease incidence. Molecular analysis confirmed PPSMV-2 as a geographically related strain of *Emaravirus toordali* and *Emaravirus cajani*. SEM revealed severe trichome deformities and collapsed stomatal guard cells in infected leaves. Biochemically, advanced disease infection increased stress injury, phenol content, peroxidase and polyphenol oxidase activities while depleting chlorophyll and soluble sugars. This reflects triggered progressive physiological impairment and active biochemical defenses. Fourier Transform Infrared Spectroscopy (FTIR) analyses revealed prominent shifts in protein, lipid and polysaccharide spectral bands (N-H, C-H, C=O, C-O-C stretching) which tentatively point to membrane damage and modified primary metabolic state following infection. Concurrently, Inductively Coupled Plasma Mass Spectrometry (ICP-MS) data demonstrated a noticeable reduction in tissue concentrations of Na, Mg, Al and K. This elemental depletion included a drop in calcium levels that may have altered downstream calcium signaling pathways and cellular structural integrity accompanied by decreased manganese concentrations that suggest a potential inhibition of manganese-activated enzymes required for carbohydrate metabolism.

**Discussion:**

Collectively, these baseline findings identify candidate spectral and mineral biomarkers unique to resistant genotypes while reinforcing the genetic dominance of PPSMV-2. This exploratory profile of SMD associated elemental and spectral shifts serves as a template for validating resistance traits across a wider range of pigeonpea genotypes.

## Introduction

Pigeon pea (*Cajanus cajan* (L.) Millsp.) is a significant grain legume grown in tropical and subtropical zones and is characterized by high protein content, resistance to adverse climatic conditions and the ability to enhance soil fertility through nitrogen fixation *via* biological mechanisms (Singh et al., 2026). The average yield of 861 kg ha^-1^ is close to its peak, though regular biotic and abiotic stress limits further growth (Shashank et al., 2025). Globally, pigeon pea is grown on 7.02 million ha with a production of 6.80 million tonnes. India contributes about 80% of world production, cultivating 4.3 mha with yearly production of 3.5 mt and a productivity of 823 kg/ha (FAOSTAT, 2024).

Sterility mosaic disease (SMD), caused by *Pigeonpea sterility mosaic emaravirus* (Family: *Fimoviridae*) is the primary economic and biological threat to pigeonpea production. SMD of pigeonpea was initially reported in 1931 by Mitra from Bihar state of India. The disease is also referred to as the “green plague” due to symptoms such as acute greenness, excessive vegetative growth, bushy stunting and sterility of floral structures, ultimately leading to severe yield losses (Sayiprathap et al., 2024). India’s economy incurs more than US$300 million in yearly losses due to this disease and under epidemic conditions, yield loss in susceptible varieties reaches 100% (Datta et al., 2026).

The eriophyid mite *Aceria cajani* is the only known vector of the disease and is a semi-persistent host of the parasite. At present there are 33 species of Emaraviruses belonging to *Fimoviridae* family of negative sense RNA viruses among which two viruses, *Pigeonpea sterility mosaic virus 1* (PPSMV-1; *Emaravirus cajani*) and *Pigeonpea sterility mosaic virus 2* (PPSMV-2; *Emaravirus toordali*) cause sterility mosaic disease in pigeonpea. Mechanistically, PPSMV-1 induces distinct chlorotic ring spots on leaves whereas PPSMV-2 induces severe mosaic mottling, leaf stunting and bushy vegetative growth. In terms of genomic differences PPSMV-1 has a five-segmented RNA genome lacking RNA6 whereas PPSMV-2 possesses a six-segmented RNA genome that uniquely includes the RNA6 fragment encoding a P27 protein of unknown function. Recent findings have shown that PPSMV- 2 is the most common species in most of the pigeonpea producing regions in India, frequently present in mixed infections with PPSMV-1 and highly conserved in terms of genomics with recombination signatures (Sayiprathap et al., 2024). Although this conservation enables the successful molecular diagnostics, it also raises concerns regarding the emergence of more virulent strains due to the recombination or mutation process.

The virus, its carrier, the environment and their complex interactions determine the epidemiology of SMD. PPSMV- 2 is not seed borne, unlike most plant viruses and the process of disease transmission primarily depends on the dynamics of vectors and their availability, the presence of an infectious substance and climatic conditions *viz*., temperature, humidity, rainfall and speed of the wind (Raj et al., 2024). High temperatures and wind speed are conducive to the growth and spread of mites while rainfall prevents the disease development. The interactions underscore the need to combine host resistance with ecological knowledge to be able to manage the diseases.

The most sustainable and environment friendly approach to dealing with SMD is the resistance of host plants. Large scale screening campaigns that have used over 13,000 pigeonpea germplasm accessions have retrieved few stable resistant lines in response to the substantial effect of pathogen diversity and genotype x environment x vectors interaction on the expression of resistance (Kandarkar et al., 2023). In addition to traditional methods of resistance screening there is growing evidence indicating that a viral infection results in massive metabolic and physiological reprogramming of the host plant. It has been reported that PPSMV- 2 infection causes a decrease in chlorophyll level, photosynthetic activity, carbohydrate metabolism and rise in phenolics and oxidative stress levels (Jiang et al., 2025). Such alterations can be seen as the transition of primary metabolism to the defense-related mechanisms which can be associated with the neglect of plant growth and reproductive development.

Besides metabolic changes, nutrient imbalance has become a crucial yet poorly studied aspect of the plant-virus interaction. The alterations in the essential and non-essential elements namely zinc, boron, nickel and cadmium may determine the severity of the disease and the predisposition to it (Jeevanraj et al., 2025). Ultra-sensitive quantification of macro and micronutrients with the inductively coupled plasma mass spectrometry (ICP-MS) enables the study of ionomic profiles comprehensively. Even though the techniques have been effective on other plant-pathogen systems, their use in pigeonpea PPSMV- 2 interactions is still in its infancy (Sharma et al., 2025).

New possibilities in the dissection of these complex interactions are given by recent developments in analytical technologies. Fourier transform infrared (FTIR) spectroscopy facilitates fast and non-destructive biochemical characterization by observing changes in functional groups of proteins, lipids and carbohydrates which characterize a metabolic fingerprint of stress responses (Langyan et al., 2026).

Despite advances in molecular diagnostics and resistance screening the integrated role of metabolic and ionomic reprogramming in determining SMD resistance still remains underexplored. The PPSMV-2 infection induces coordinated disruptions in primary metabolism and elemental homeostasis which together govern disease susceptibility and resistance outcomes. The PPSMV- 2 infection triggers an oxidative burst accompanied by suppression of catalase activity resulting in uncontrolled reactive oxygen species (ROS) accumulation that damages cellular components. This oxidative stress triggers electrolyte leakage and disrupts calcium and potassium homeostasis impairing active ion transport mechanisms. In addition, the virus redirects host lipids to form quasi-spherical membrane bound structures (100–150 nm in diameter) compromising original membrane integrity. These physiological disruptions trigger a severe loss of chlorophyll and proline, culminating in the stunting and sterility characteristic of the ‘Green Plague’ (Elbeaino et al., 2015).

The present study employed a multidimensional approach to examine sterility mosaic disease in pigeonpea. This involved combining field phenotyping with molecular confirmation, analyzing physiological and biochemical alterations through enzyme assays, characterizing molecular level spectral changes through FTIR and profiling the elemental composition of infected leaf tissues using ICP-MS.

## Materials and methods

### Field evaluation of genotypes against sterility mosaic disease

#### Experimental design and plant material

Field evaluations were conducted during the *Kharif* 2022 and *Kharif* 2023 cropping seasons at the Research Farm, Department of Plant Pathology, Chaudhary Charan Singh Haryana Agricultural University (CCS HAU) Hisar, Haryana, India (29.1°N, 75.7°E; altitude 215 m). The experimental site has a semi-arid subtropical climate with hot summers and cold winters. During, *Kharif* 2022 cropping season, twenty-five pigeonpea breeding genotypes were screened while thirty-three genotypes were evaluated during *Kharif* 2023 cropping season. The susceptible genotype ICP 8863 was included as SMD check in both the years. The experimental design used a randomized block layout (RBD) with two replications. Each plot measured 4 m × 3 m with row spacing of 75 cm and plant spacing of 25 cm.

#### Inoculation and disease scoring

Artificial inoculation was performed on 12–15 days old seedlings (two to three leaf stage) using the leaf stapling method to facilitate transmission of *Pigeon pea sterility mosaic emaravirus* (PPSMV- 2) by the eriophyid mite *Aceria cajani*. Leaf-stapling method for transmission of PPSMV was followed as per the protocol described by (Pande et al., 2012). Young SMD infected leaflets collected in a moist cloth bag were observed for mite infestation under a binocular microscope to ensure a minimum of 10 mites per leaf. The mite-infested leaflets were then stapled onto test plants at the two to three leaf stage in such a way that the undersurface of the diseased leaflet comes in contact with both surfaces of the leaf of the test plant to anchor mites for transfer and their feeding results in PPSMV transmission onto the test plant.

Plants were observed regularly and SMD symptoms were recorded at both vegetative and flowering stages. Disease incidence was calculated using the formula:


$$\%\text{ SMD incidence}=\frac{\text{No}.\text{ of SMD infected Plants}}{\text{ Total number of plants observed }}\times 100$$

Based on disease incidence values the evaluated genotypes were systematically classified into four distinct resistance categories to facilitate comparative assessment. Genotypes exhibiting an incidence of ≤10% were categorized as resistant, indicating a high level of tolerance against the disease. Those with incidence values ranging from 10.1% to 20% were considered moderately resistant, reflecting partial tolerance. Genotypes showing disease incidence between 20.1% and 40% were grouped as susceptible suggesting a considerable level of vulnerability. Finally, genotypes with incidence exceeding 40% were classified as highly susceptible (Nene et al., 1981) indicating severe disease impact and minimal resistance. This classification framework was adopted in accordance with previously established criteria (Sharma et al., 2015).

#### Seasonal consistency and weather data

In order to assess the stability of resistance over two cropping season’s data were subjected to analysis of variance (ANOVA) for each corresponding year. Meteorological data *viz*., maximum and minimum temperature (°C), morning and evening relative humidity (%), rainfall (mm), wind speed (km h^−1^) and sunshine duration (hours) were obtained from the CCS HAU observatory (**Supplementary Material Table 1**). The data were also analyzed to assess the relationship between environmental factors and the development of disease.

### Molecular characterization of PPSMV- 2

#### RNA extraction

The symptomatic leaf samples of pigeonpea were collected from genotypes identified as susceptible and moderately susceptible during field screening. The total RNA was extracted from these samples using the QIAGEN RNeasy^®^ Plant Mini Kit (QIAGEN, Germany) according to the manufacturer’s recommendations. The quality of RNA was checked by agarose gel electrophoresis and the purity was confirmed by determining the A260/A280 ratio using a NanoDrop 8000 Spectrophotometer (Thermo Fisher Scientific, USA). The RNA samples were stored at -20 °C.

#### Reverse transcription PCR

The RNA was then reverse-transcribed into cDNA by means of the SuperScript™ III First-Strand Synthesis SuperMix Kit (Invitrogen, USA). Specific primers for the RNA-2 and RNA-3 segments of PPSMV-1 and PPSMV- 2 (**Table 1**) were employed for the amplification of the target DNA (Sayiprathap et al., 2024). The procedure for the amplification of the target DNA by means of specific primers for the RNA-2 and RNA-3 segments of PPSMV- 2 follows Elbeaino et al. (2015). The 25 μL of the PCR mixture consisted of 2.5 μL of 10x buffer, 3.0 μL of MgCl_2_ (25 mM), 0.5 μL of dNTPs (10 mM each), 0.5 μL of each specific primer, 0.5 μL of Taq DNA polymerase (Thermo Scientific, Lithuania) and 1 μL of cDNA. The cycling conditions for the amplification of the target DNA consisted of 4 minutes at 94 °C, 35 cycles of 92 °C for 1 minute, 57 °C for 30 seconds, 72 °C for 45 seconds and a final extension at 72 °C for 5 minutes. Amplicons were resolved on 1.2% agarose gels (TAE buffer) stained with ethidium bromide and visualized using a gel documentation system.

**Table 1:** Synthetic oligonucleotide primers utilized for polymerase chain reaction (PCR) amplification of PPSMV.

Virus	Segment	Primer sequence (5 ‘ — 3 ‘)	Amplicon size
*Pigeonpea sterility mosaic virus 1* (PPSMV-1)	RNA-2	GATGGTCTAGTAATTAGTTTGAG	392
		CTCTATGTGCTTATGTCCAGCA	
	RNA-3	ACATAGTTCAATCCTTGAGTGCG	322
		ATATTTTAATACACTGATAGGA	
*Pigeonpea sterility mosaic virus 2* (PPSMV- 2)	RNA-2	GACTTACATGATTATTGCTCCA	384
		TGTCATATGATCACTATCTGTA	
	RNA-3	GAGAGTAGTGAGTTGGAACCGAT	284
		GAGTATCCCAGCAGCCATTATT	

#### Sequencing and phylogenetic analysis

The representative PCR amplicons were purified and sequenced using a Sanger method involving dideoxy nucleotides (Eurofins Genomics, India). The nucleotide sequences were analyzed using a BLASTN tool for similarity searches with the NCBI nucleotide database (https://blast.ncbi.nlm.nih.gov). The sequences were aligned using a multiple sequence alignment tool, MUSCLE (Edgar, 2004) and Phylogenetic analysis of PPSMV-2 isolates was done by using Neighbor-Joining (NJ) method. To assess phylogenetic relationships 1,000 bootstrap replicates were conducted. Further validation of analysis was done by additional PPSMV-2 sequences retrieved from GenBank and subjected to multiple sequence alignment tool ClustalW, in MEGA11 software (Tamura et al., 2021).

### Microscopy and structural analysis

#### Scanning electron microscopy

Representative three leaf samples from three plants of *Cajanus cajan* (cv. ICP 8863) with *Aceria cajani* along with three healthy leaf samples from three plants of ICPL 99090, were collected early in the morning at 06:00 h to minimize turgor-related distortions and the samples were collected with sterile tools under humid conditions to preserve cellular integrity (Pathan et al., 2008). Samples were fixed in 2.5% glutaraldehyde prepared in 0.1 M phosphate buffer (pH 7.2) for 4 h at 4 °C (Lopez-Llorca and Duncan, 1988). After fixation tissues were dehydrated through a graded ethanol series (30-100%) and subjected to critical point drying. Dried specimens were mounted on aluminum stubs with conductive carbon tape and sputter-coated with ~20 nm of gold before imaging with a JEOL JSM-6390LV scanning electron microscope operated at 1.0 kV. For higher resolution ultra-structural observations of symptomatic and asymptomatic leaf samples were examined using field-emission scanning electron microscopy (FESEM). More than five imaging fields were analyzed per sample. This dual SEM/FESEM strategy facilitated comparative visualization of mite colonization and disease-induced surface changes.

#### Biochemical assays

Standard protocols were followed to compare the biochemical profiles of healthy/asymptomatic and SMD infected pigeonpea leaves at the flowering stage. The specific methodology is detailed below.

#### Chlorophyll content

Chlorophyll content was quantified using 100 mg of finely chopped leaf samples placed in a tube containing 10 ml dimethyl sulfoxide (DMSO) and incubated overnight. An estimation of chlorophyll content of pigeonpea leaves was made using Wellburn and Lichtenthaler (1984) method. The following formula was used to calculate the chlorophyll extracted in DMSO at 480, 645 and 663 nm.


$$\text{Chlorophyll a}=(12.19\times {\text{A}}_{663}–3.45\times {\text{A}}_{645})\times \frac{\text{Volume}}{1000\times \text{Weight}}$$

$$\text{Chlorophyll b}=(21.19\times {\text{A}}_{645}–5.32\times {\text{A}}_{663})\times \frac{\text{Volume}}{1000\times \text{Weight}}$$

Total Chlorophyll (mg/g)=20.2(OD645)+8.02(OD663)×(V/(1000×wt))

### Total soluble sugars

#### Extraction of total soluble sugars

Total soluble sugars were extracted following the method described by Dubois et al. (1956). A 100 mg of the powdered sample was transferred into a 100 mL flat-bottomed volumetric flask. The sample was extracted with 10–20 mL of 80% ethanol by heating in a water bath at 70 °C for 1 hour. The supernatant was filtered and collected in a 25 mL volumetric flask. This extraction procedure was repeated five to seven times until the residue was completely free of sugars and the filtrates were pooled.

#### Estimation of total soluble sugars

For quantification 1 mL of the sugar extract was diluted with 9 mL of distilled water. 1 mL aliquot of this diluted extract was transferred to a test tube, followed by the addition of 2 mL of 2% phenol solution. Subsequently 5 mL of concentrated sulphuric acid was added rapidly and directly into the liquid surface to ensure efficient mixing.

The test tubes were gently agitated and allowed to cool at room temperature for 30 minutes. The absorbance of the reaction mixture was measured at 490 nm using a spectrophotometer. A standard curve was prepared concurrently using glucose. The total soluble sugar content was calculated from the standard curve and expressed as mg glucose equivalents per gram of fresh weight.

### Total phenolic content

#### Extraction of phenolic compounds

A 500 mg sample was crushed in 5 mL of 80% (v/v) methanol and transferred to a Falcon tube. The mixture was centrifuged at 10,000 rpm for 10 minutes and the supernatant was collected. The remaining residue was re-extracted with an additional 5 mL of 80% (v/v) methanol and centrifuged under identical conditions. Both supernatants were pooled to estimate the total phenolic content (TPC).

#### Estimation of total phenolic content

The total phenolic content was determined according to the method described by Bray and Thorpe (1954). One milliliter of the plant extract was mixed with 7.5 mL of distilled water in a test tube. Diluted Folin-Ciocalteu reagent (1 N) was added and the mixture was shaken thoroughly. Subsequently, 1 mL of saturated sodium carbonate solution was added and the final volume was adjusted to 10 mL with distilled water. The reaction mixture was incubated for 1 hour at room temperature before measuring the absorbance at 725 nm using a spectrophotometer. A reagent blank was prepared by substituting the sample extract with distilled water. The total phenolic content was quantified using a concurrent standard curve of gallic acid and expressed as mg gallic acid equivalents per gram of fresh weight (mg GAE g^−1^ fresh weight).

#### Relative stress injury

Electrolyte leakage (EL) was determined as per method of Sullivan and Ross (1979). 100 mg fresh leaf segments (5 mm) were incubated in 10 mL of distilled deionized water at 32 °C for 2 hours. Initial electrical conductivity (EC1) was measured using a CM-115 conductivity meter (Kyoto Electronics, Kyoto, Japan). Samples were then autoclaved at 121 °C for 20 minutes to achieve complete tissue lysis and electrolyte release. Following cooling to 25 °C, final electrical conductivity (EC 2) was recorded and EL was calculated using formula (EL = (EC1/EC 2) x 100).

#### Peroxidase activity

Peroxidase (POX) activity was evaluated according to the method described by Maehly (1954) with minor modifications. The reaction was performed using guaiacol as hydrogen donor. The assay mixture contained a 20 mM guaiacol solution, phosphate buffer and crude enzyme extract. The enzymatic reaction was initiated by adding hydrogen peroxide H_2_O_2_. The progressive oxidation of guaiacol to tetraguaiacol was tracked by recording the linear increase in absorbance at 470 nm over a 3 minute interval against an enzyme-free blank control.

#### Polyphenol oxidase

Analysis of PPO activity was determined following the method described by Gauillard et al. (1993). The homogenization of the leaf samples (0.5 g) was done in 5 ml of sodium phosphate buffer (0.1 M, pH 6.5). After centrifugation, the resulting supernatant was used as an enzyme source. The reaction was started by adding 0.4 ml catechol (0.01 M), 3.0 ml of sodium phosphate buffer (0.1 M, pH 6.5) and 0.4 ml of enzyme extract. The results were noted as changes in absorbance at 495 nm and articulated as changes in the O.D. min^−1^ g^−1^ fresh weight.

#### Fourier transform infrared spectroscopy

FTIR spectroscopy was conducted on leaf samples of healthy and SMD-infected plants using a Nicolet iS50 FTIR spectrometer (Thermo Fisher Scientific, India) in attenuated total reflectance mode. To further compare FTIR spectra were recorded using a Nicolet iS 50 FTIR spectrometer using ATR mode equipped with diamond crystal accessory. The FTIR was conducted for the leaf samples of 33 pigeonpea genotypes screened during *Kharif* 2023 and the spectra were recorded over a range of 4000–400 cm^−1^ with 32 co-added scans for each sample with a resolution of 2–4 cm^−1^. OMNIC software was used for baseline correction, vector normalization and smoothing of spectra. Student’s t-test was conducted using R software to statistically evaluate the observed differences in mean wavenumbers between the healthy and infected samples and confirm significance level.

### Inductively coupled plasma mass spectrometry

#### Sample preparation

Leaf tissue from healthy/asymptomatic plants (ICPL 99090) and SMD-infected plants (ICP 8863) at flowering stage were subjected to washing with deionized water, followed by a brief rinse with 0.1% Triton X-100 solution and later oven dried. About 200 mg of dried leaf tissue was weighed and then digested with 4 mL concentrated nitric acid (HNO_3_, 65%) and 1 mL hydrogen peroxide (H_2_O_2_, 30%) in Teflon digestion vials in a microwave digestion system, Multiwave Pro (Anton Paar, Austria), at 170-200 °C for 40–60 minutes. After digestion, the solution was allowed to cool and then diluted to 50 mL with ultrapure water. The solution was then filtered through 0.2 µm PTFE syringe filters and kept at 4 °C until further use.

#### ICP-MS analysis

Ionomic profile analysis of the solution was carried out in ICP-MS (iCAP RQ, Thermo-fisher Scientific India) equipped with a cross-flow nebulizer and autosampler. High-purity argon served as both the carrier and plasma gas. Instrument calibration was performed using ICP multi-element standard solutions (Sigma-Aldrich) and quality assurance was ensured through the use of reagent blanks and certified reference materials. (**Supplementary Material Figures 1A–E**). ICP-MS analysis was executed on microwave-digested composite samples using triplicate technical replicates, with subsequent statistical significance evaluated via one-way analysis of variance (ANOVA). The elements analyzed included Na, Ca, Mg, Al, K, Fe, Mn, Ni, Zn and Cd. Final concentrations were expressed in μg/kg. Data are expressed as fold change. A dumbbell plot was generated in R v4.6.0 using the ggplot2 package (Wickham, 2016) to visualize differences in elemental concentrations between healthy and PPSMV-2 infected pigeonpea leaves.

## Results

### Field screening of pigeonpea genotypes for SMD

**Figure 1:**
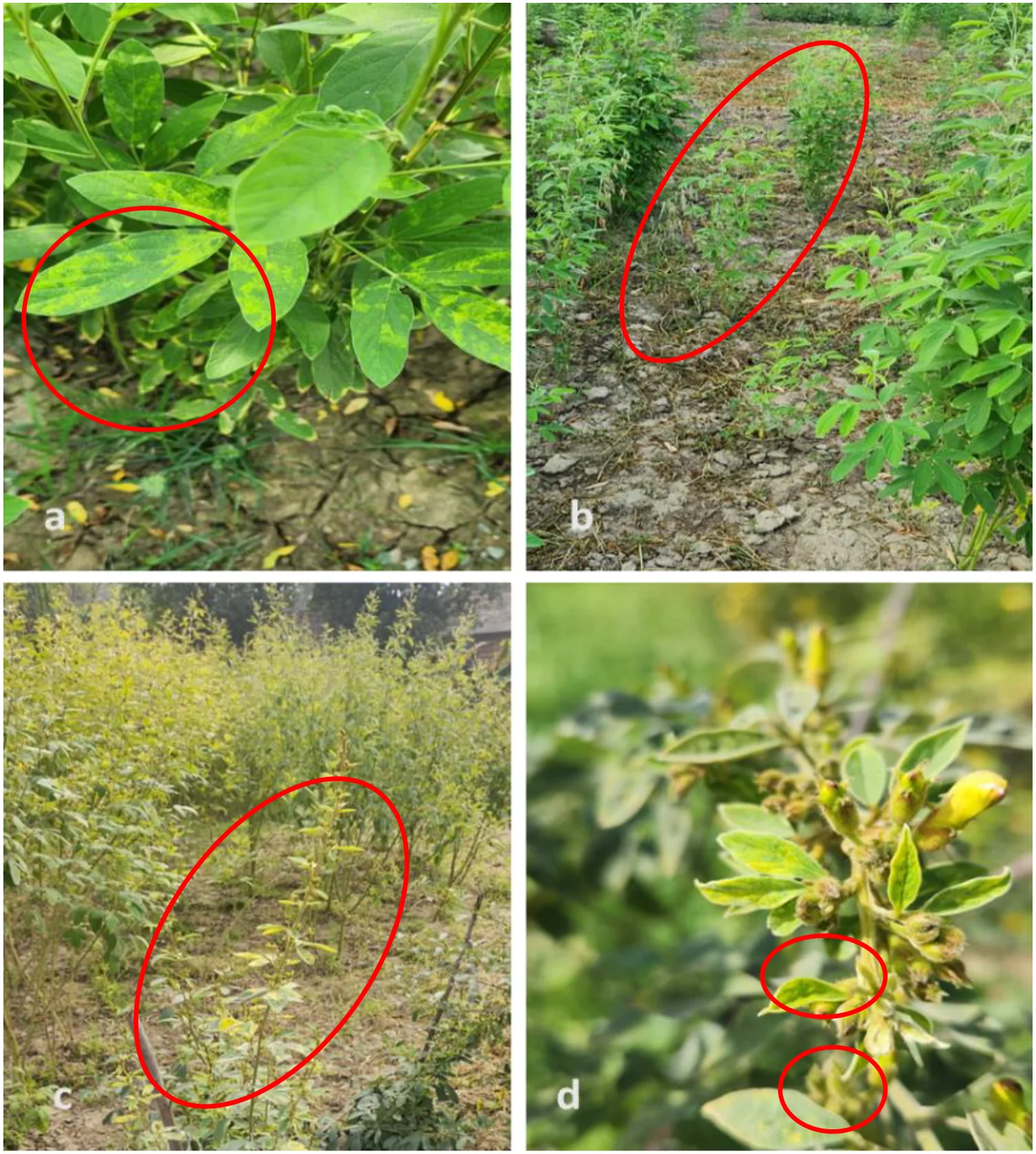
Representative symptoms of sterility mosaic disease (SMD) in pigeonpea **(a)** characteristic mosaic pattern on leaves **(b)** pronounced stunting of infected plants **(c)** complete sterility accompanied by reduced leaf size and malformed foliage **(d)** absence of flowering in severely infected plants.

**Figure 2:**
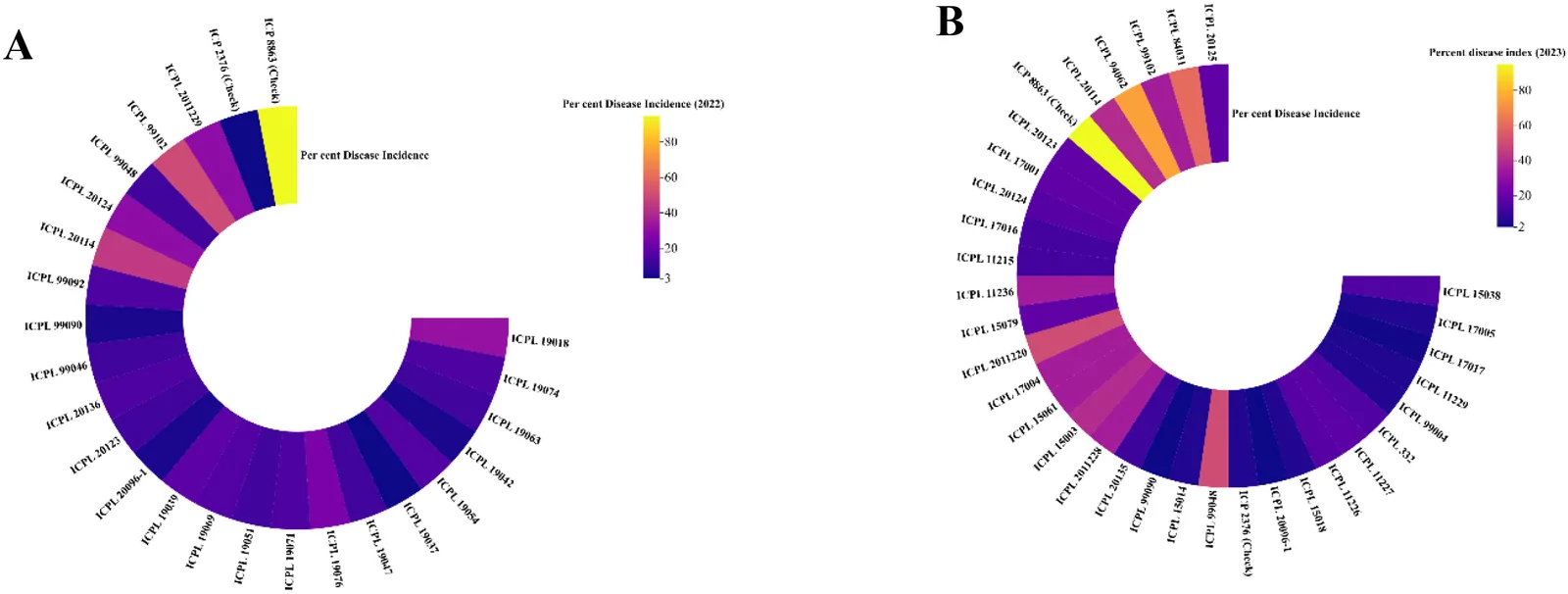
**(A)** Response of pigeonpea genotypes to sterility mosaic disease during *Kharif* 2022. **(B)** Response of pigeonpea genotypes to sterility mosaic disease during *Kharif* 2023.

Field screening over two seasons revealed prominent differences among pigeonpea genotypes in response to SMD (**Figure 1** Characteristic disease symptoms of SMD and **Figures 2A, B**). Response of pigeonpea genotypes to SMD during *Kharif* 2022 & 2023 respectively). During *Kharif* 2022, among 25 tested genotypes, four entries (ICPL 20096-1, ICPL 19037, ICPL 19042 and ICPL 99090) were resistant. The resistant check ICP 2376 remained symptom free. ICPL 20114 proved to be the most susceptible genotype with the maximum disease incidence while susceptible check ICP 8863 recorded the highest disease incidence showing its extreme susceptibility. Most genotypes were found to be moderately resistant, showing partial tolerance. The ANOVA results revealed that the disease incidence was highly significant (Fp <0.001) demonstrating a strong differentiation between the genotypes screened with a least significant difference (LSD _0.05_) of 5.45 and a low coefficient of variation (CV = 10.90%) for *Kharif* 2022 screened genotypes (**Supplementary Material Table 2A**).

During *Kharif* 2023, evaluation of 33 genotypes confirmed the stability of resistance in nine genotypes namely ICPL 15018, ICPL 15014, ICPL 17017, ICPL 17005, ICPL 99004, ICPL 20096-1, ICPL11229, ICPL20135 and ICPL99090 exhibiting complete resistance. ICPL 20096–1 and ICPL 99090 emerge as promising genotypes for resistance breeding during both the seasons. Similarly, the ANOVA results for *Kharif* 2023 screening revealed highly significant variations in disease incidence (*Fp* < 0.001), enabling a clear differentiation among genotypes. This strong variation was characterized by a least significant difference (LSD 0.05) of 7.59 and an acceptable Coefficient of Variation (CV = 18.30%) validating the reliability of the trial (**Supplementary Material Table 2B**).

**Figure 3:**
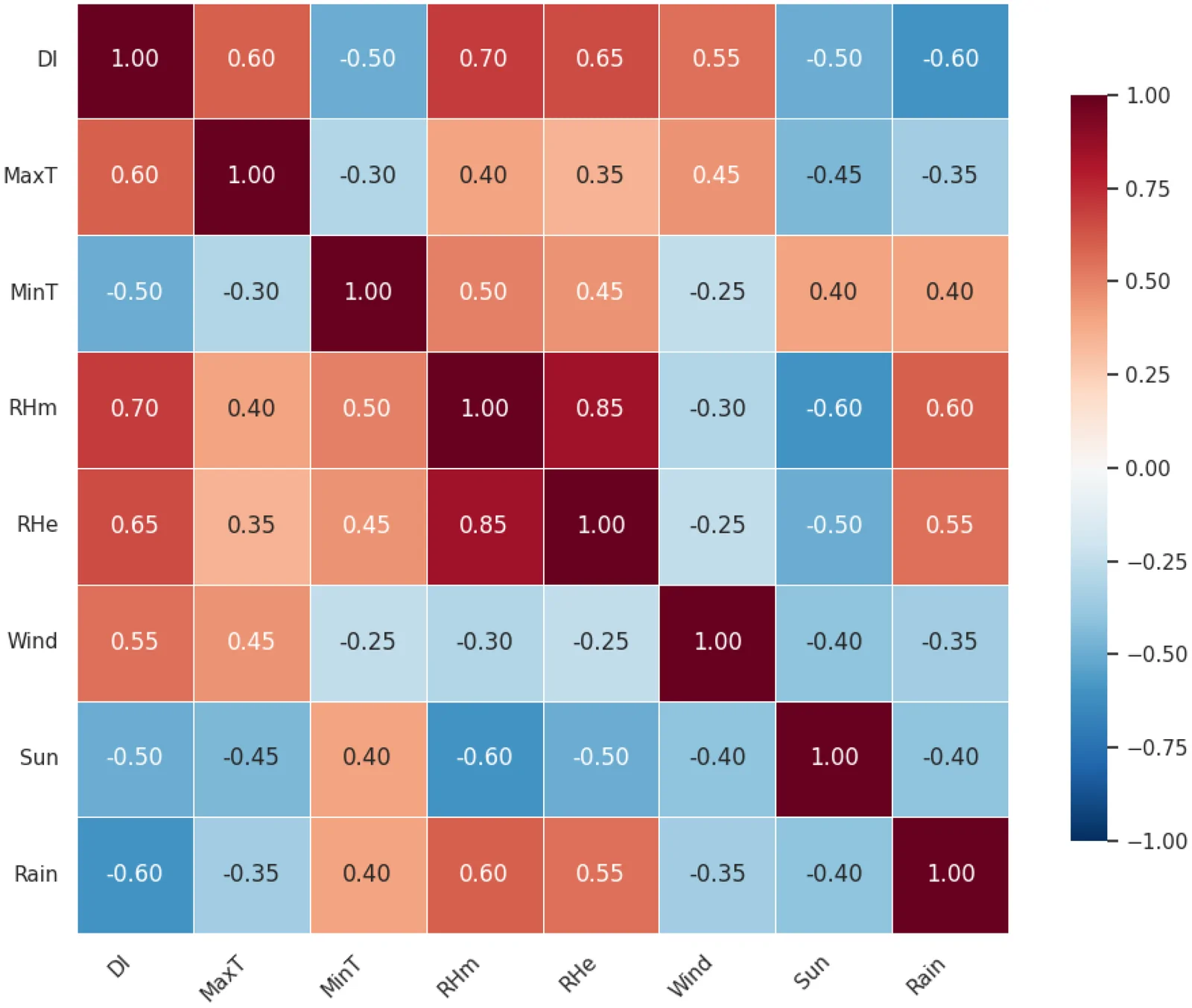
Correlation heatmap illustrating relationship among environmental variables and sterility mosaic disease (SMD) incidence. Positive correlations are indicated by warmer tones, while negative correlations are represented by cooler tones. D.I, Percent disease incidence; MaxT (°C), maximum temperature; MinT (°C), minimum temperature; RHm(%)., morning relative humidity; RHe(%)., evening relative humidity;s Wind velocity(km/hr); Sunshine(hrs/day); Rainfall(mm).

The influence of environmental factors on SMD incidence is evident from the correlation heat map (**Figure 3**). Maximum temperature and wind velocity demonstrated positive associations with disease incidence hence indicating favorable conditions for vector activity and disease spread. In contrast, minimum temperature and sunshine hours exhibited negative relationship with SMD incidence suggesting reduced epidemic development under cooler conditions and longer sunlight exposure. Both morning and evening relative humidity displayed strong positive associations with disease incidence highlighting the role of humid conditions in enhancing mite proliferation. Rainfall showed a negative association likely due to its disruptive effect on vector populations. These observations collectively emphasize the importance of genotype x environment × vector interactions in shaping SMD epidemiology.

### Molecular characterization confirms PPSMV- 2 dominance

Molecular analysis confirmed the exclusive presence of *Pigeonpea sterility mosaic emaravirus2* (PPSMV-2) in all symptomatic samples. RT-PCR assays consistently amplified the expected fragments of 384 bp (RNA-2) and 284 bp (RNA-3) among all the symptomatic/infected samples during both the seasons. No amplification corresponding to healthy control/asymptomatic samples and PPSMV-1 was observed. Representative 13 samples & 5 samples gel pic is presented in (**Supplementary Material Figures 2A, B**). These findings indicate that PPSMV- 2 is predominant viral species associated with SMD across the studied location and two cropping seasons demonstrating its widespread distribution and dominance in the field. Sequencing data deposited under accession numbers PQ782228 and PV604122 validated the dominance of PPSMV-2 within the population.

### Phylogenetic analysis of pigeonpea sterility mosaic virus

**Figure 4:**
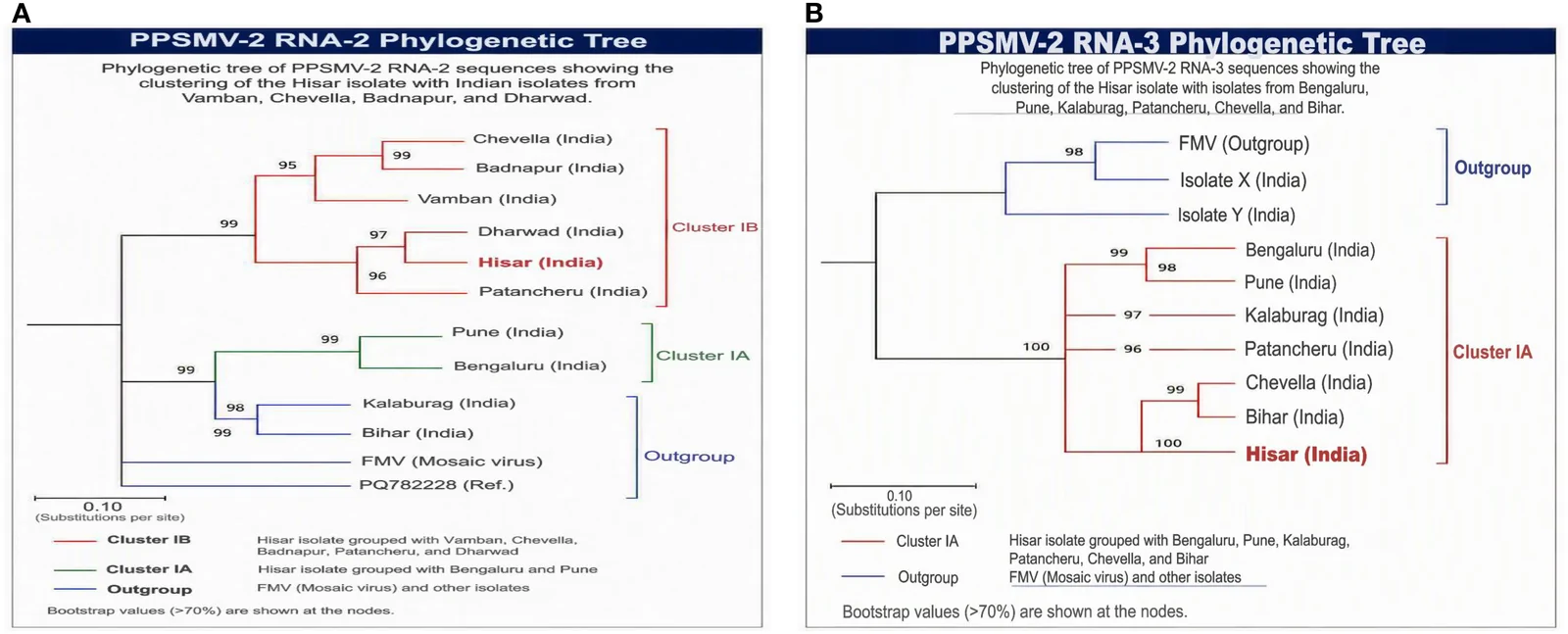
**(A)** Phylogenetic tree of PPSMV- 2 RNA-2 sequences & **(B)** Phylogenetic tree of PPSMV- 2 RNA-3 sequences.

The evolutionary relationship of Hisar isolate was investigated using Neighbor-Joining (NJ) method. Hisar isolate showed high genetic similarity and a close evolutionary association with the *Emaravirus toordali* isolate (Patancheru-BRS1117). It shares a common lineage with *Emaravirus toordali* isolates from Vamban, Bengaluru and Gulbarga. The observed short branch lengths indicate low genetic divergence and high conservation within the RNA-2 segment. Fig mosaic virus (FMV) serving as an out group, formed a distinct branch with a longer branch length confirming its distant evolutionary relationship (**Figure 4A**).

Similarly, Neighbor-Joining (NJ) phylogenetic tree constructed using RNA 3 sequences demonstrated that Hisar isolate grouped within the same clade with *Emaravirus cajani* and various pigeonpea sterility mosaic virus isolates. Strong associations were noted with *Emaravirus cajani* isolates from Kalaburagi/Gulbarga and Bengaluru as well as the Patancheru isolate from Chevella. High statistical support (bootstrap values of 100, 92, and 68) confirms the reliability of these clusters. While closely related, Hisar isolate forms a separate branch within this clade highlighting specific sequence variations and genetic divergence. FMV outgroup remained distinctly separated, further validating the distinction of these Emaravirus populations (**Figure 4B**). Overall, the analysis demonstrated that Hisar isolate is a geographically related strain of *Emaravirus toordali* and *Emaravirus cajani*. The high degree of nucleotide sequence conservation across Indian populations suggests a common evolutionary origin with limited genetic variability among these viruses.

### SEM revealed tissue deformation and vector association

**Figure 5:**
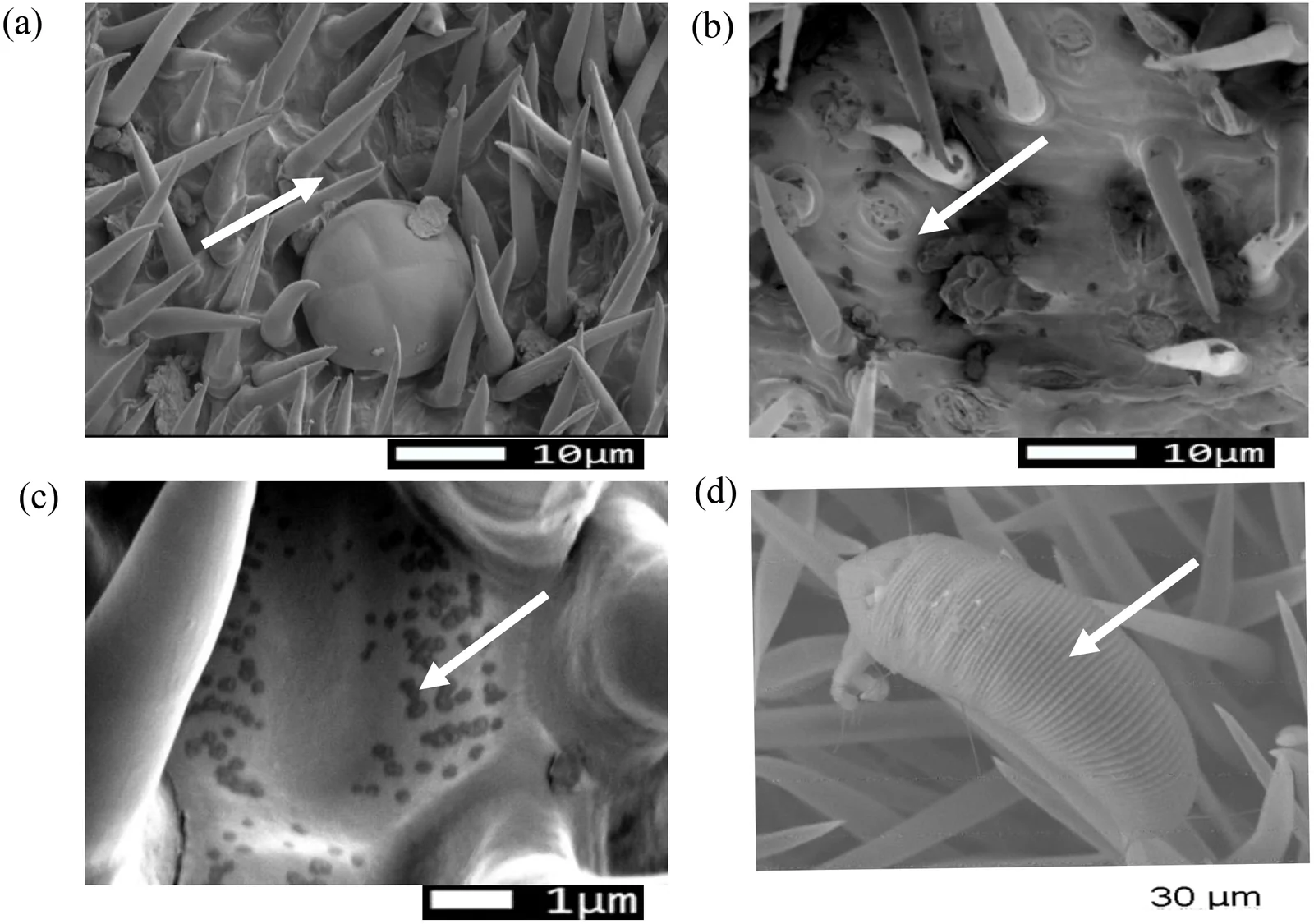
Scanning electron microscopy images of pigeonpea leaves from 45 days old plants: **(a)** Rigid, sharp trichomes on a healthy leaf (ICPL 99090) **(b)** Collapsed stomata & deformed trichomes in the infected leaf **(c)** Structural damage in the infected leaf **(d)** *Aceria cajani* mite on the infected leaf surface (ICP 8863).

Representative healthy (ICPL 99090) and PPSMV infected leaf samples from susceptible check ICP 8863 at same developmental stage (45 days old plant) collected under comparable experimental conditions were examined under identical microscopic conditions and observations were confirmed across different regions of the samples to ensure consistency of the reported structural features (**Supplementary Material Figures 3A–I**). In healthy leaves trichomes were erect, well-defined structures (**Figure 5a**), distorted trichomes and collapsed stomata in the infected leaf (**Figure 5b**) Structural damage in SMD infected leaf (**Figure 5c**) Eriophyid mite (*Aceria cajani*) on young symptomatic leaves (**Figure 5d**).

The characteristic features of adult mites included 100-200 µm length, annulated and anteriorly placed legs indicative of their feeding activity on the leaves. Although direct mechanical feeding by the mite on epidermal and underlying mesophyll cells acquiring virus only from infected cells induced minimal structural distortion, it effectively inoculated the host with PPSMV-2. Systemic colonization by PPSMV-2 resulted in profound macro-structural alterations including severe leaf crinkling, mosaic, chlorosis and reduced laminar area accompanied by widespread metabolic disruption.

**Figure 6:**
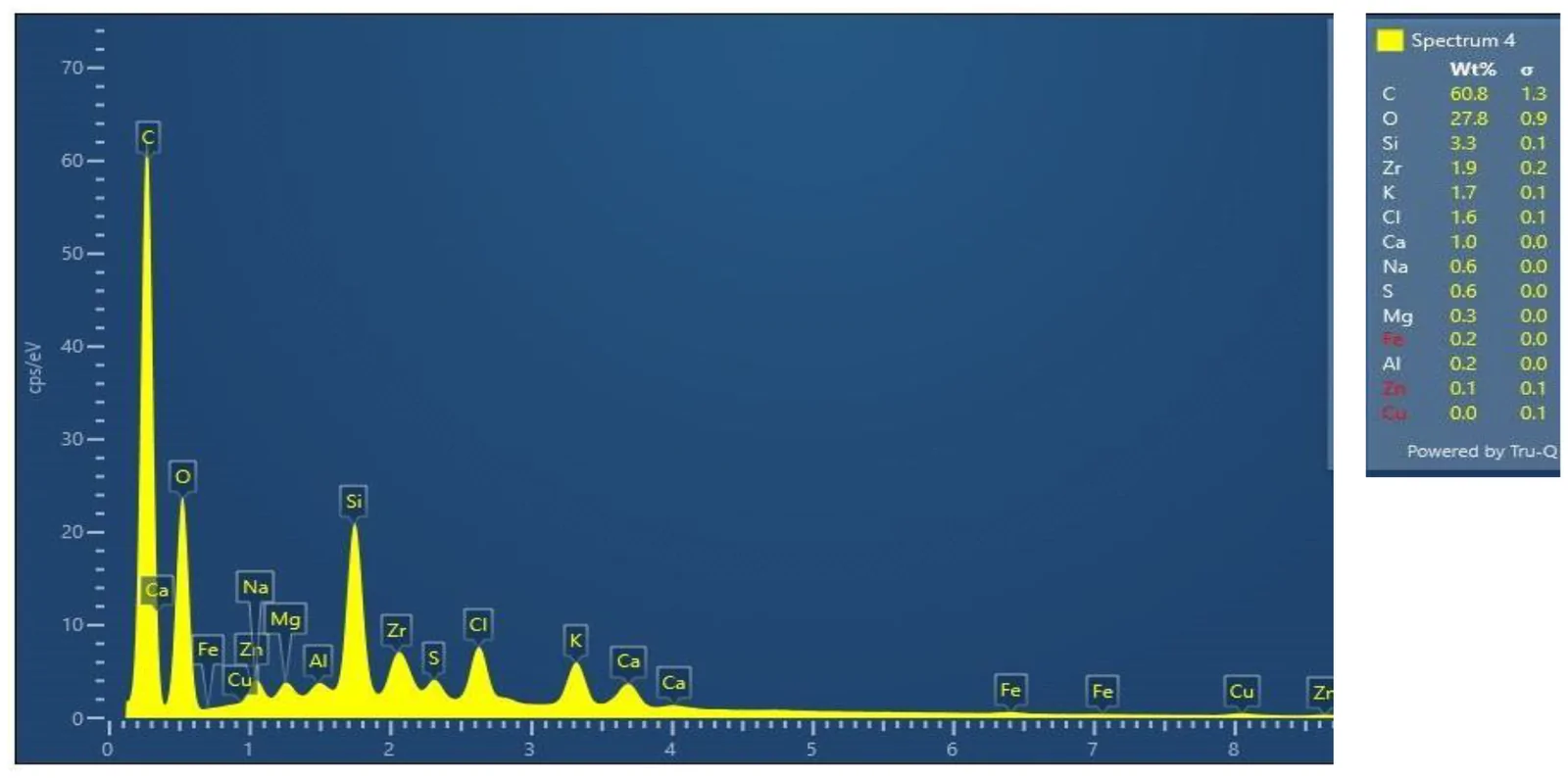
Energy-dispersive X-ray spectroscopy (EDS) profile of sterility mosaic disease (SMD)-infected pigeonpea leaves (ICP 8863 during flowering stage) showing prominent elemental peaks.

Energy Dispersive X-Ray (EDS) spectral analysis presented in (**Figure 6**) were obtained from multiple spots to achieve a representative average for acquiring spectra of elemental status using symptomatic susceptible check (ICP 8863) at 45 days old plant. The infected tissue was collected under comparable experimental conditions and multiple imaging fields were examined to ensure that the observed features were consistently reproducible across samples. The spectra revealed that carbon (60.8%) and oxygen (27.8%) were the major elements present in the leaf tissues. Importantly, a strong carbon and oxygen signal is expected in the leaf tissues as these elements form the primary structural matrix of biological tissues. Notably, the elevated carbon levels likely arise from background signals of the conductive carbon mounting tape and perhaps do not indicate true endogenous biological variations. The additional spectra for silicon, zirconium, potassium, chlorine, calcium, sodium, sulfur, magnesium, iron, aluminum, zinc and copper were observed in the infected tissue. However, level of calcium in infected leaves was found to be decreased.

### Biochemical assays

The physiological and biochemical parameters in pigeonpea plants varied markedly with reference to the disease incidence of PPSMV- 2 infection. One-way ANOVA revealed markedly differences among treatments for all parameters (P < 0.0001) **Table 2**. Relative stress injury (RSI) increased progressively with increase in disease incidence indicating enhanced cellular membrane damage under stress conditions. The RSI value increased from 10.52 in the resistant genotype to 13.80 in moderately resistant genotype, 16.94 in the susceptible genotype and reached maximum (21.83) in highly susceptible genotype. Further, one-way ANOVA demonstrated a highly considerable treatment effect (F_3_,_16_ = 242.9, P < 0.0001) suggesting that disease progression substantially aggravated stress injury in pigeonpea plants. In contrast, chlorophyll content (CC) declined consistently with increasing disease incidence reflecting impaired photosynthetic efficiency. 

**Table 2:** Summary of one-way ANOVA for physiological and biochemical parameters.

Parameter	df (Treatment, error)	F-value	P-value
Relative Stress Injury (RSI)	(3, 16)	242.90	<0.0001
Chlorophyll Content	(3, 16)	55.47	<0.0001
Total Phenol Content	(3, 16)	75.51	<0.0001
Total Sugar Content	(3, 16)	165.70	<0.0001
Peroxidase (POD) Activity	(3, 16)	366.00	<0.0001
Polyphenol Oxidase (PPO) Activity	(3, 16)	134.80	<0.0001

**Figure 7:**
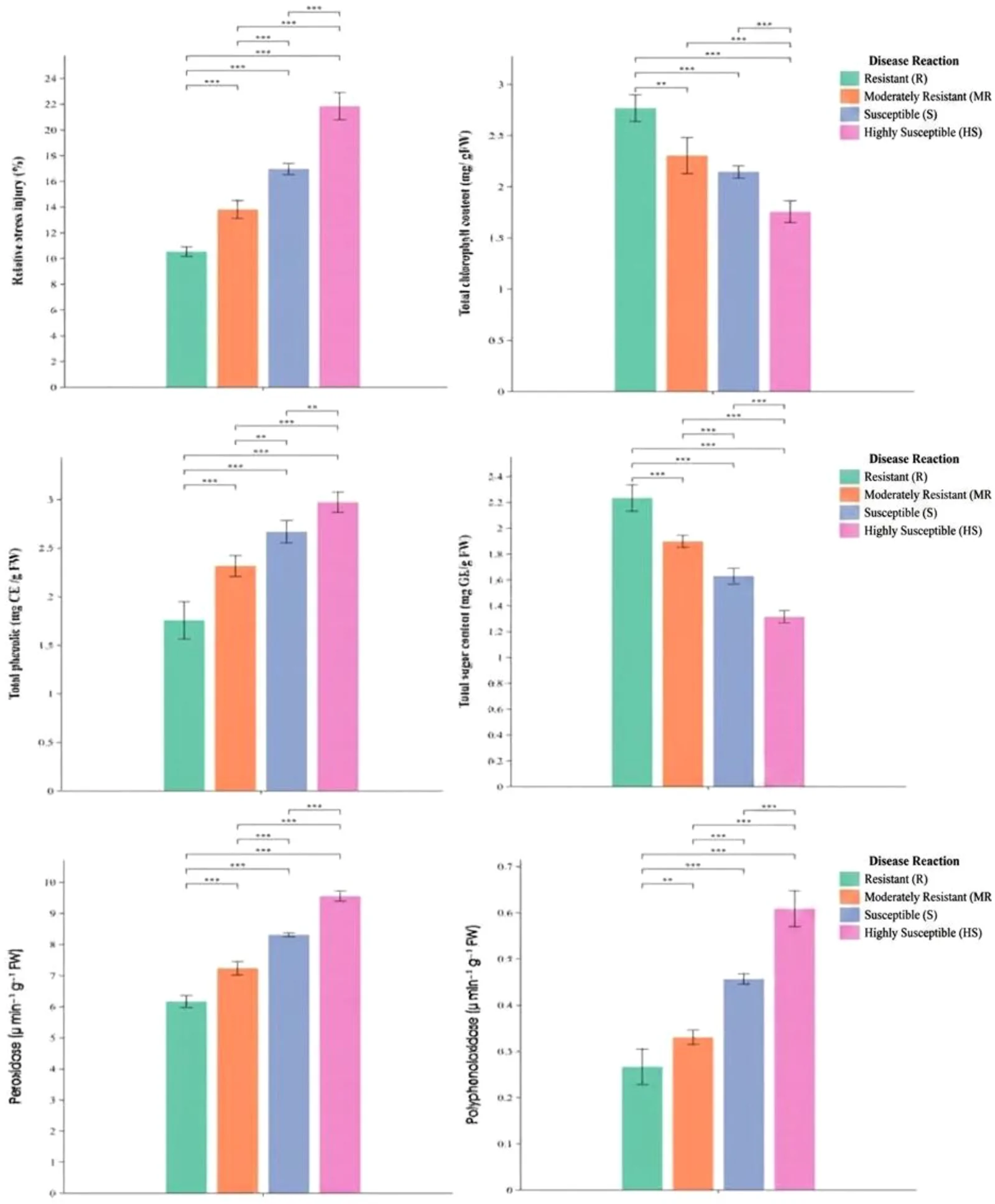
Physiological and biochemical alterations in pigeonpea following PPSMV- 2 infection across disease reaction (Resistant 0-10%; Moderatey resistant 10.1-20%;Susceptible 20.1-40%;Highly susceptible >40.1%): **(A)** relative stress injury, **(B)** total chlorophyll content, **(C)** total phenolic content, **(D)** total soluble sugars, **(E)** peroxidase activity and **(F)** polyphenol oxidase activity illustrating progressive metabolic and stress-associated responses with increasing disease infection. Error bars represent the standard error of the mean (±SE). Horizontal brackets spanning across treatment bars indicate pairwise comparisons; asterisks denote the level of statistical significance (** p < 0.01, *** p < 0.001).

Plants of the resistant genotype exhibited highest chlorophyll content (2.77) which decreased to 2.30, 2.14 and 1.75 in moderately resistant, susceptible and highly susceptible genotypes respectively. The differences among disease reaction were noticeable (F_3_,_16_ = 55.47, P < 0.0001) indicating progressive deterioration of the photosynthetic apparatus under severe infection. Total phenol content showed a gradual increase with disease infection suggesting activation of defense related secondary metabolism. Phenol content increased from 1.75 in the resistant genotype to 2.31 in moderately resistant genotype, 2.67 in susceptible genotype and peaked at 2.97 in the highly susceptible genotype. ANOVA confirmed conclusive variation among disease reaction (F_3_,_16_ = 75.51, P < 0.0001) exhibiting an induced biochemical defense response under increasing stress. Similarly, total sugar content exhibited a decreasing trend with increasing disease infection. The highest sugar content (2.23) was recorded in the resistant genotype which gradually decreased to 1.90, 1.63 and 1.31 in moderately resistant, susceptible and highly susceptible genotypes respectively. Statistical analysis revealed markedly differences (F_3_,_16_ = 165.7, P < 0.0001) suggesting metabolic disruption and reduced carbohydrate accumulation in severely affected plants. The activity of peroxidase (POD) increased substantially with disease infection and hence enhanced antioxidant defense. POD activity increased from 6.15 in resistant genotype to 7.23 in moderately resistant genotype, 8.30 in susceptible genotype and 9.55 in highly susceptible genotype. ANOVA indicated a distinct treatment effect (F_3_,_16_ = 366.0, P < 0.0001) showing strong induction of oxidative stress responsive enzymes during disease progression. Likewise, polyphenol oxidase (PPO) activity exhibited a progressive increase with increasing disease infection. PPO activity rose from 0.266 in resistant genotype to 0.330 in the moderately resistant genotype, 0.456 in susceptible genotype and reached 0.608 in the highly susceptible genotype. The differences were highly noticeable (F_3_,_16_ = 134.8, P < 0.0001) indicating activation of enzymatic defense mechanisms associated with phenolic oxidation and pathogen resistance (**Figures 7A–F**).

Overall, increased disease infection was associated with a distinct increase in RSI, phenol content, POD and PPO activities whereas chlorophyll and sugar contents declined highlighting progressive physiological impairment and activation of defense related biochemical mechanisms in response to disease stress.

### FTIR spectral analysis

**Figure 8:**
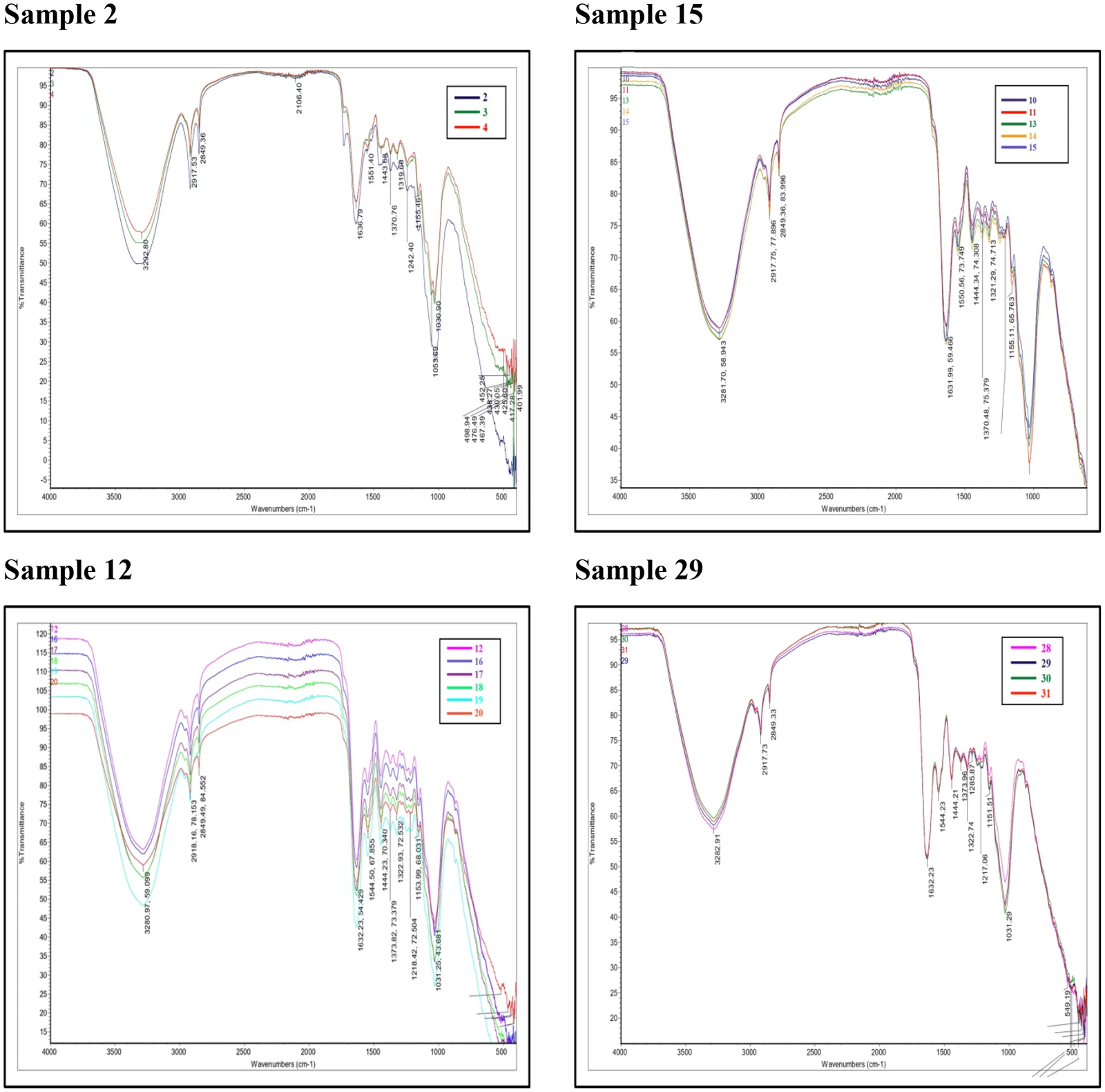
Representative FTIR spectra of biological replicates obtained from healthy *viz.*, Sample-2 (ICPWSMD2202) and 15 (ICPWSMD2215) and PPSMV infected pigeonpea *viz.*, Sample-12 (ICPWSMD2212) and 29 (ICPWSMD2229) leaf tissues. Multiple independently collected replicate samples were analyzed under identical experimental conditions. The overlaid spectra demonstrate the reproducibility of major spectral features and the consistency of infection-associated biochemical alterations across biological replicates.

In the present study, the FTIR technique has been employed as an additional analytical tool to study infection in the plant tissue samples. Among all the 33 FTIR spectra obtained for pigeonpea leaves from genotypes screened during *Kharif* 2023 (**Supplementary Material Figure 4**) eighteen samples were selected for a more detailed comparison based on the observed characteristic shifts in the spectrum. Two spectra were obtained from a representative healthy plants namely sample 2 (ICPWSMD2202) and sample 15 (ICPWSMD2215) supported by replicates and similarly, two spectra were obtained from SMD infected plants namely sample 12(ICPWSMD2212) and sample 29 (ICPWSMD2229) including the replicates (**Figure 8**) Healthy samples showed well-defined absorption bands at~3330 cm^−1^ (O-H/N-H stretching), ~2925 cm^−1^ (C-H stretching of lipids), ~1640 cm^−1^ (amide I, protein). In contrast, the infected samples showed changes. A considerable increase in intensity of the O-H stretching region (3300–3400 cm^−1^) indicated the formation of hydrogen bonds, which might be related to the phenolic compounds. Moreover, the shift in Amide I band at 1640 cm^−1^ and the Amide II band at 1540 cm^−1^ indicate the spectral shifts consistent with changes in protein/lipid secondary structures possibly, the denaturation of the proteins (**Supplementary Material Table 3**). Furthermore, the increase in intensity/spectral shifts in the fingerprint region related to carbohydrates (1200–900 cm^−1^) indicate remodeling of the cell wall during the infection. Independent t-tests confirmed highly significant shifts (p < 0.01) for Amide I (1635.83) to (1633.07 cm^-1^); (t = 3.255), (p = 0.0045), C-H banding (1320.83) to (1322.72cm^-1^); (t = -4.2), (p = 0.0011), and C-O-C bands (1040.41) to (1030.73cm^-1^); (t = 3.85), (p = 0.0039). Significant changes (p < 0.05) were obtained in the O-H/N-H bonding (3305.27) to (3282.42cm^-1^); (t = 2.317), (p = 0.0454) and Amide II bands (1507.74) to (1546.09cm^-1^); (t = -2.293), (p = 0.0475). Conversely, the C-H stretching band showed a borderline non-significant change, closely approaching statistical significance (t = 2.045, p = 0.0558). Independent *t*-tests confirmed that five out of the six analyzed functional groups changed significantly following infection (**Table 3**).These preliminary spectral alterations/trends require further high-throughput quantitative validation.

**Table 3:** FTIR band assignments, mean wavenumbers, and independent sample *t*-test comparisons between healthy and PPSMV-2 infected pigeonpea leaves.

S. no.	Band assignment -wave number (cm^−1^)	Mean (healthy) cm^-1^	Mean (infected) cm^-1^	T-calculated	P-value	Significance level
1	O-H/N-H (~3330) (hydrogen bonding, phenolics, proteins)	3305.27	3282.42	2.317	0.0454	*
2	C-H stretching (~2925) (lipids, carbohydrates)	2917.88	2917.7	2.045	0.0558	NS
3	Amide I (~1640) (C=O stretching of proteins)	1635.83	1633.07	3.255	0.0045	**
4	Amide II (~1540) (N-H bending and C-N stretching of proteins)	1507.74	1546.09	-2.293	0.0475	*
5	C-H Bending (~1410) phenolic O-H	1320.83	1322.72	-4.2	0.0011	**
6	C-O-C (~1050) (polysaccharides, cell wall components)	1040.41	1030.73	3.85	0.0039	**

* Significant at 5% level P≤ 0.05; ** Significant at 1% level P≤ 0.01; NS, Non Significant p > 0.05.

### Ionomic imbalances detected by ICP-MS

**Figure 9:**
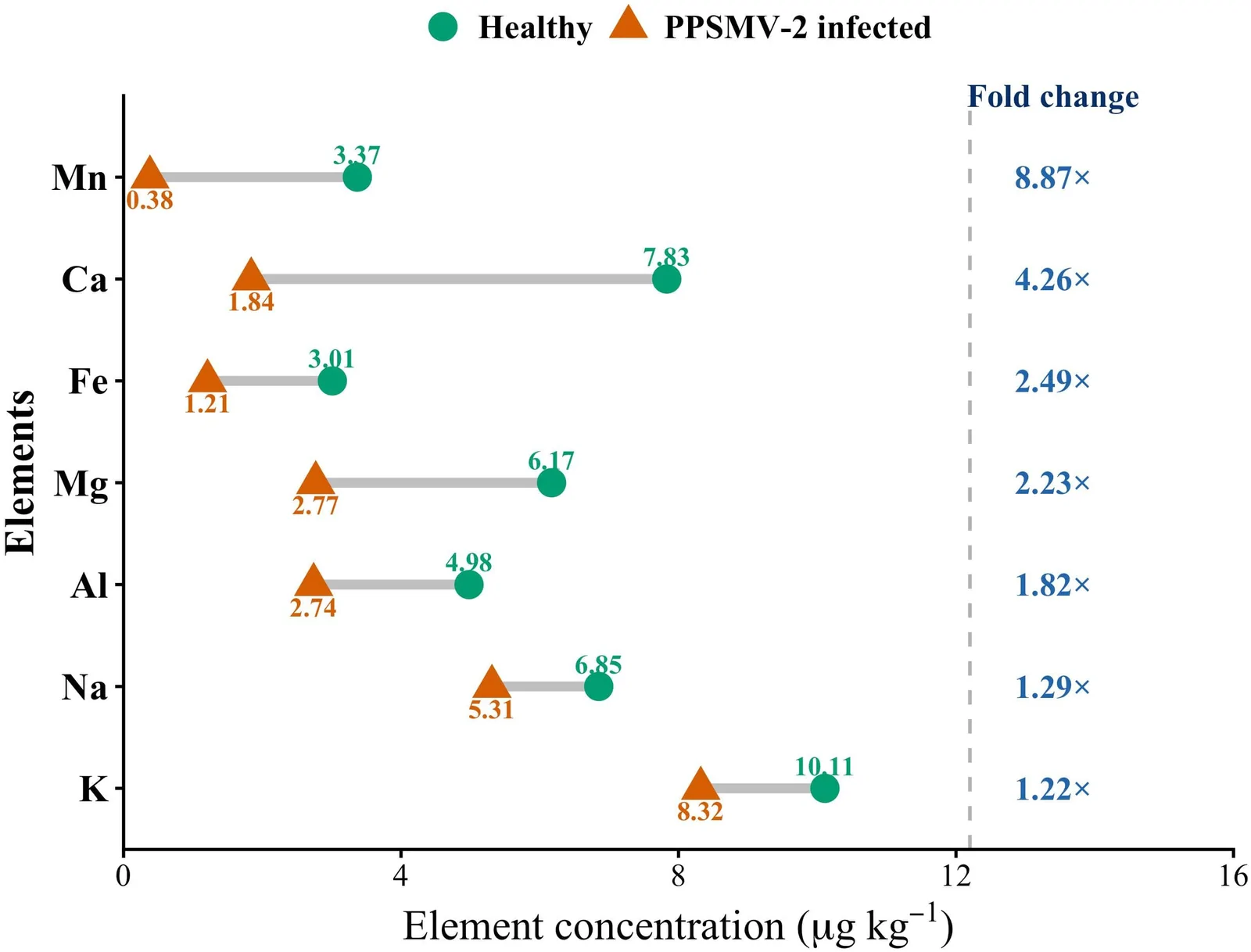
ICP-MS elemental dumbbell plot showing relative differences in elemental concentrations (μg/kg) in pigeonpea leaves (healthy ICPL 99090 *vs* infected ICP 8863).

Ionomic profiling using ICP-MS revealed markable alterations in elemental compositions within infected tissues. Healthy plants exhibited a relatively balanced nutrient composition whereas infected plants showed a marked depletion of essential macronutrients. One-way analysis of variance (ANOVA) validated the high statistical significance (**Table 4**) and precision of the observed ionomic alterations (P = 0.05). The absolute mean differences between healthy and infected tissues for all tested elements exceeded their respective critical difference (CD) values, confirming that the observed elemental depletions are highly significant. Data reliability was further supported by low absolute standard deviations (SD ≤ 0.21) and minimal coefficients of variation (CV < 3%) across all replicates. Marked reductions in absolute concentrations were observed for macronutrients including potassium (K; from 10.11 to 8.32 μg/kg, 1.21-fold decrease), sodium (Na; from 6.85 to 5.31 μg/kg, 1.29-fold decrease), magnesium (Mg; from 6.17 to 2.77 μg/kg, 2.22-fold decrease) and aluminum (Al; from 4.98 to 2.74 μg/kg, 1.81-fold decrease). Notably, calcium (Ca) content decreased markedly 4.25 fold from 7.83 μg/kg in healthy tissue to 1.84 μg/kg in infected samples. Trace element analysis revealed noticeable decline specifically iron (Fe; from 3.01 to 1.21 μg/kg, 2.48-fold decrease) and manganese (Mn; from 3.37 to 0.38 μg/kg, 8.86-fold decrease) signaling a possibility of potential disruption of critical metabolic and enzymatic processes (**Figure 9**). Conversely, heavy metals accumulated in infected tissues; nickel (Ni) and cadmium (Cd) concentrations rose to 0.47 μg/kg and 0.60 μg/kg respectively from levels below the limit of detection (< LOD) in healthy control (**Supplementary Material Figure 5**, **Supplementary Material Table 4**).The observed ionomic changes, specifically calcium (Ca) depletion and the accumulation of heavy metals like Cadmium (Cd) and Nickel (Ni) are primarily secondary stress responses rather than direct viral targeting of those specific ions. In combination with observed reductions of chlorophyll in bio chemical studies. The results also indicate a possible link between viral infection, impaired photosynthetic performance and changes in nutrient homeostasis during disease development. However, further targeted studies are required to establish the mechanistic relationship between these physiological, ionomic changes and the progression of SMD.

**Table 4:** Summary of one-way ANOVA for elemental composition (ICP-MS).

Element	Healthy leaves (Mean)	Infected leaves (Mean)	CD (P = 0.05)	CV (%)	SD (n=3)
Na	6.85	5.31	0.53	2.48	0.15
Mg	6.17	2.77	0.46	2.94	0.13
Al	4.98	2.74	0.37	2.73	0.11
K	10.11	8.32	0.74	2.30	0.21
Ca	7.83	1.84	0.36	2.13	0.10
Fe	3.01	1.21	0.18	2.41	0.05
Mn	3.37	0.38	0.15	2.35	0.04

## Discussion

Pigeonpea sterility mosaic virus (PPSMV- 2) is species of genus Emaravirus and the causal agent of sterility mosaic disease (SMD) of pigeonpea (*Cajanus cajan* (L.) Millsp.) The disease known as ‘green plague’ causes the infected plants to remain in the vegetative state without flower production. SMD has been reported from India and a few other South-East Asian countries and has resulted in significant yield loss in pigeonpea production (Patil and Kumar, 2015).

This article aims to provide a comprehensive understanding of SMD through a combination of parameters including field screening, molecular diagnosis, ultrastructural, biochemical, spectroscopic and ionomic studies. Of particular interest is the identification of stable resistance and plant stress responses to PPSMV- 2 infection. ICPL 20096–1 and ICPL 99090 genotypes are found to be resistant to SMD during field screening for two consecutive years. The resistant genotypes obtained in the present study are in line with the findings of the long-term resistance of ICRISAT breeding lines (Sharma et al., 2015; Sayiprathap et al., 2022) thereby reinforcing their reproducibility in terms of resistance response across multiple seasons highlights the reliability and robustness of the field screening methodology employed in the present investigation. The identified genotypes serve as valuable genetic resources for location specific resistance breeding in pigeonpea.

RT-PCR and sequencing results confirmed the presence of the primary causal agent in the symptomatic plants of pigeonpea. Phylogenetic analysis of the RNA 2 sequences shares a common lineage with *Emaravirus toordali* isolates from Vamban, Bengaluru and Gulbarga. For RNA 3 sequences strong associations were noted with *Emaravirus cajani* isolates from Kalaburagi/Gulbarga and Bengaluru as well as the Patancheru isolate from Chevella. Such genetic stability of the virus has also been observed in the past (Elbeaino et al., 2015; Kumar et al., 2017).The Fig mosaic virus (FMV) outgroup remained distinctly separated further validating the distinction of these emaravirus populations.​​The high degree of nucleotide sequence conservation across Indian populations suggests a common evolutionary origin with limited genetic variability among the Indian emaravirus populations. Although this low variation ensures accurate diagnosis it simultaneously increases the probability of the spread of a virulent mutation across India.

SEM analysis of the infected leaf tissues showed noticeable tissue deformation including the deformed trichomes and stomata. This affects the mechanical and gaseous exchange functions of the leaf and increases the stress on the host plant. This stress on the host plant due to the mechanical effects of mite and metabolic stress caused by the virus is likely to cause a rapid progression of the disease in the host plants. Such interactions between the mite and the virus have been reported in cereals and figs (Amrine and Stasny, 1994; Elbeaino et al., 2015).The presence of *Aceria cajani* mite on the infected tissues further supports the interaction between the mite and the host plant and hence the transmission of the virus (Kumar et al., 2003).The transmission of PPSMV- 2 and the occurrence of mixed infections in pigeonpea are consistent with the previous findings (Rohini et al., 2025). In addition, the ability of *A. cajani* to disseminate through wind currents facilitates viral spread to newly emerging plants. The vector dispersal is passive, primarily aided by wind currents with the mites blown up to 2 km from the source of inoculums (Reddy et al., 1990). The strong correlation between environmental factors and disease occurrence underscores the importance of climate smart disease forecasting and vector management strategies. High humidity in particular favors disease development while rainfall inhibits disease occurrence possibly through its adverse effect on mite populations. High humidity and moderate temperatures likely favor mite survival, reproduction and feeding activity thereby enhancing viral transmission whereas rainfall may suppress disease development by limiting mite dispersal and survival (Pallavi et al., 2020). Previous studies conducted by Muniyappa and Nangia (1982) demonstrated that resistance in pigeonpea cultivars is closely associated with the prevalence of the eriophyid mite *Aceria cajani* highlighting the important role of the vector in the epidemiology and spread of sterility mosaic disease. This is important considering that SMD epidemics are largely influenced by vector and climatic conditions (Kumar et al., 2003).

Further, the biochemical assays indicated chlorophyll content (CC) declined consistently with the increasing disease infection reflecting impaired photosynthetic efficiency. Resistant plants exhibited the highest chlorophyll content which decreased with increasing disease incidence indicating progressive deterioration of photosynthetic apparatus under severe infection. Concurrently, the total phenol content showed a gradual increase with disease infection while the antioxidant enzyme activities i.e. POX and PPO were highly activated in the susceptible genotypes suggesting activation of defense related secondary metabolism. Such biochemical responses in the susceptible genotypes were earlier reported in pigeonpea under SMD infection (Zhao et al., 2016) as well as in other legume virus pathosystems which confirmed the modulation of oxidative stress as an integral component of the plant’s defense mechanism (Walters, 2003; Hull, 2014). PPO is involved in the oxidation of monophenols and o-diphenols to form more toxic o-quinones compared to their phenolic precursors which can directly inactivate viral proteins and inhibit the replication machinery. The increase in PPO activity in response to PPSMV- 2 stress therefore points towards a biochemically active stress response mechanism mediated by phenolic oxidation and reinforcement of resistance in hosts. These results therefore show that infection by PPSMV- 2 causes oxidative stress in plants as well as mobilizes phenolic compounds and antioxidant systems in plants. It appears that regulation of primary metabolism, stress damage and enzymatic stress response systems is a major factor in determining tolerance in pigeonpea against sterility mosaic disease. Furthermore, resistant genotypes trigger an early high intensity spike in POX activity to catalyze the rapid cross-linking of cell wall phenols. Based on the previous literature, we further hypothesize that this in muro lignification creates a physical barrier or restriction that triggers programmed cell death, effectively starving the virus of living host tissue. In susceptible plants, these responses are too delayed or weak to prevent the virus from becoming systemic and siphoning nutrients Rathi et al. (1986); Elbeaino et al. (2015) on Emaravirus host-defense. Similarly, total sugar content exhibited a decreasing trend with increasing disease infection suggesting metabolic disruption and reduced carbohydrate accumulation in severely infected plants. It is hypothesized from the previous findings that carbon resources are redirected toward PPSMV-2 replication leading to a probable increase in reducing sugars at the expense of host nitrogen storage proteins. The virus significantly reduces proline levels, compromising the plant’s osmo-protectant capacity and disrupting its nitrogen homeostasis during chlorosis (Prameela et al., 1989).

Reactive oxygen species (ROS) and reactive nitrogen species (RNS) act as dual-edged swords (Ali et al., 2025) functioning as both essential signaling molecules and cellular damage agents. Their biosynthesis is compartmentalized within chloroplasts, mitochondria and peroxisomes to maintain tight concentration control. Adaptation relies on spatiotemporal signaling dynamics where the timing and location of reactive species determine the physiological outcome. A critical mechanistic feature is the crosstalk between ROS and RNS which modulates plant immunity and responses to abiotic cues. These species regulate adaptation to diverse stressors including pathogens drought and salinity by influencing signal transduction pathways. Redox signaling is integrated with hormonal and epigenetic networks to build long-term stress resilience. Optimal plant health requires a precise balance between the production and scavenging of these reactive molecules. Advanced imaging techniques now allow for the *in vivo* visualization of these dynamics within redox signaling pathways. This framework highlights the importance of molecular specificity in gene regulation during environmental stress. Understanding these interactions is vital for unraveling the mechanisms of plant growth and environmental resilience.

Our study further suggests the exploratory hypothesis for the plant’s defense mechanism in the context of SMD infection in pigeonpea which has been correlated with the spectroscopic and ionomic data. The FTIR technique has been utilized to further characterize the infected plant tissue with results indicating the broadening of the O-H stretching region, weakening of the amide I and II bands and elevated carbohydrate fingerprint region. This may be attributed to the accumulation of phenolics and lignin-related polymers which are well-documented defense metabolites induced during fungal and viral pathogenesis (Mahlein, 2016). The weakening of amide I and amide II bands reflects the degradation of structural and pathogenesis-related (PR) proteins consistent with protease activity observed during host-pathogen interactions. The increase in the carbohydrate fingerprint region corresponds to altered polysaccharide metabolism potentially reflecting pectin demethylation, callose deposition or changes in cell wall carbohydrate composition as a part of the host defense response (Mahlein et al., 2018). Lipid peroxidation-associated changes may also contribute to spectral shifts in the 2800–3000 cm^−1^ region reflecting membrane integrity loss in infected tissues. The FTIR technique has been successfully employed in patho-systems involving wheat rust, potato late blight etc (Naumann, 2000, 2006) but the study on the interaction in the pigeonpea PPSMV- 2 pathosystem is new and we present these correlated spectroscopic and ionomic variations as an exploratory hypothesis for the pigeonpea defense mechanism against SMD.

Hasegawa et al., 2012 reported that ionomic profiling using ICP-MS indicated a noticeable imbalance in the elemental content of the infected tissues. There was an accumulation of Zn, Ni and Cd which have been reported to cause ROS, interfere with enzyme activity and affect cellular metabolism. The proposed findings align with the previous literature suggesting that such depletion disrupts calcium-dependent protein kinases (CDPKs), potentially compromising the host’s primary defense signaling pathways. Furthermore, published evidence confirms that the loss of Ca^+2^ breaks down pectin cross-linking *in muro* which compromises cell wall structural integrity and facilitates viral spread. The resulting ionic imbalance possibly impairs stomatal regulation, leading to the transpiration failure and severe stunting symptoms in the “Green Plague.” Zn over accumulation, though an essential micronutrient, can affect enzyme activity and hormonal homeostasis. The preliminary findings indicate that PPSMV-2 infection could alter aspects of primary metabolism while potentially disrupting nutrient uptake and transport mechanisms which may induce stress in the plant. Consequently, this exploratory work seeks to provide an initial framework connecting ionomic shifts with SMD progression. PPSMV- 2 infection exploits cellular heterogeneity where distinct cell types such as epidermis, mesophyll and phloem exhibit unique defense, resource and viral replication responses, moving beyond uniform tissue-level reactions (Ali et al., 2026). Key stress-response regulation mechanisms include spatiotemporal reactive oxygen species (ROS) regulation, cell-type-specific ionomic imbalances and a mosaic pattern of hormonal crosstalk (JA/SA) driven by viral p5/p6 proteins, effectively hijacking cellular resources. Similarly, Bera et al. (2022) demonstrated that potyviral 6K1 protein stability is dynamic, increasing over time during Turnip mosaic virus (TuMV) infection. Reduced stability of ectopically expressed 6K1 is driven by post-transcriptional modifications and altered protein degradation pathways.

EDS may serve to substantiate the localized accumulation of elements in infected tissues. Salt et al. (2008) proposed that combining FTIR, ICP-MS and EDS analyses could offer analytical framework for SMD assessment by providing metabolic and ionomic biomarkers to support conventional screening methods. In the present study EDS was utilized as a preliminary, semi-quantitative indication to observe elemental surface distribution, which requires further validation by more robust quantitative methods.

Consequently, these findings indicate a potential avenue where resistance breeding is complemented by ecological management. Future investigations would benefit from exploring functional roles of individual phenolics, antioxidant enzymes and elemental distributions to better understand the mechanisms behind resistance. Leveraging integrated physiological, biochemical and omics platforms could provide valuable insights to facilitate gene discovery and marker development.

## Conclusion

Integrating field, molecular, structural and biochemical observations provides preliminary insights into SMD resistance. The stable genotypes ICPL 20096–1 and ICPL 99090 reveal a strong potential for location-specific resistance breeding. Initial physiological comparisons indicate a divergent defense response between host phenotypes. Resistant genotypes appear to employ rapid activation of peroxidase and polyphenol oxidase potentially establishing an immediate physical barrier to restrict viral progress. Conversely, susceptible plants display acute chlorophyll depletion and severe systemic metabolic disruption. Furthermore, ICP-MS and FTIR baseline findings offers a preliminary systems-level framework for examining the interconnected biochemical, structural, spectroscopic and ionomic variables involved in SMD resistance. Future studies can build on this evidence to pursue targeted molecular dissection. This exploratory model suggests that a future integrated multi-omics workflow combining transcriptomics, metabolomics and ionomics could effectively map host regulatory networks and isolate specific candidate genes governing SMD tolerance.

## Data Availability

The datasets presented in this study can be found in online repositories. The names of the repository/repositories and accession number(s) can be found in the article/**Supplementary Material**.

## Supplementary material

Supplementary Table 1 Seasonal weather parameters recorded during field trials.

Supplementary Figure 1 ICPMS- elemental calibration. **(A)** Calibration curves for elemental analysis: representative calibration curves obtained via ICP-MS for target isotopes ^107^Ag, ^111^Cd, ^115^In, ^137^Ba, ^205^Ti & ^208^Po in STD mode. Each panel displays the linear regression equation f(x), coefficient of determination (R^2^), background equivalent concentration (BEC) and limit of detection (LoD) across a concentration range from 0 to 250 ppb. Signal intensity is plotted as counts per second (cpsx10^4^). **(B, C)** Representative calibration curves obtained via ICP-MS for target isotopes ^27^Al ^39^K, ^209^Bi & ^44^Ca in STD mode. Each panel displays the linear regression equation f(x), coefficient of determination (R^2^), background equivalent concentration (BEC) and limit of detection (LoD) across a concentration range from 0 to 250 ppb. Signal intensity is plotted as counts per second (cpsx10^4^). **(D, E)** Representative calibration curves obtained via ICP-MS for target isotopes ^23^Na ^24^Mg, ^55^Mn & ^57^Fe in STD mode. Each panel displays the linear regression equation f(x), coefficient of determination (R^2^), background equivalent concentration (BEC) and limit of detection (LoD) across a concentration range from 0 to 250 ppb. Signal intensity is plotted as counts per second (cpsx10^4^).

Supplementary Table 2 **(A)** Screening of pigeonpea genotypes against sterility mosaic disease during *Kharif* 2022. **(B)** Screening of pigeon pea genotypes against sterility mosaic disease *Kharif* 2023.

Supplementary Figure 2 RT-PCR detection of PPSMV- 2 in infected pigeonpea leaf samples using RNA-2 (384 bp) and RNA-3 (284 bp) specific primers. **Figure (2A)** lane M: 100 bp DNA ladder; lanes 1–14 infected field samples; **Figure (2B)** lane M: 100-bp DNA ladder; lanes 1–5 infected field samples.

Supplementary Figure 3 **(A–D)** SEM image representing the biological replicates of infected pigeon pea leaves used to assess surface structures, highlighting a dense distribution of non-glandular trichomes surrounding central particulate or cellular structures. The image confirms consistency in surface topography and structural integrity across independent biological replicates. Scale bar = 10 µm. **(E, F)** SEM image representing a biological replicate of healthy **(E)** infected **(F)** pigeon pea leaves used to assess surface structures, highlighting a dense distribution of non-glandular trichomes surrounding central particulate or cellular structures. The image confirms consistency in surface topography and structural integrity across independent biological replicates. Scale bar = 10 µm. **(G, H)** **(G)** Scanning electron micrograph (1000x) capturing a detailed view of the surface micro-morphology of the pigeon pea leaves. The imaging field reveals an agglomeration of spherical micro-particles embedded within an amorphous matrix, demonstrating the local surface texture and structural arrangement of the sample. Scale bar = 10 µm. **(H)** Low-magnification SEM imaging field of the pigeon pea leaf tissue. Wide-field scanning electron micrograph (45x) illustrating the macro-level surface topography and overall tissue architecture. This imaging field demonstrates the homogeneous distribution and dense coverage of micro-structures across a broad surface area of the biological specimen. Scale bar = 100 µm. **I** Overview scanning electron micrograph (25x) displaying a broad macroscopic field of view across the pigeon pea leaf surface. The imaging field captures a major leaf vein running vertically alongside net-like minor venation networks, demonstrating the uniform distribution of surface microstructures and an isolated surface feature or deposit in the upper right quadrant.

Supplementary Figure 4 Fourier-transform infrared spectroscopy (FTIR) spectra of 33 genotypes evaluated during *Kharif* 2023 showing percentage transmittance across wavenumbers from 4000 to 400 cm^−1^ along with key characteristic absorption bands including a broad peak around 3335 cm^−1^ (O–H stretching), C–H stretching bands near 2917 cm^−1^ and distinctive fingerprint region peaks (including 1731, 1636, and 1031 cm^−1^ corresponding to the functional group.

Supplementary Table 3 FTIR band assignments and biochemical interpretations of spectral shifts in representative healthy (ICPL 99090) and PPSMV-2 infected (ICP8863) pigeonpea leaves.

Supplementary Figure 5 **(A)** ICP-MS raw concentration and intensity data report of infected pigeon pea leaves (ICP 8863) generated via Qtegra software (Thermo Scientific iCAP RQ) displaying elemental concentrations (ppb), average signal intensities (counts per second, cps) and relative standard deviations (RSD) for analyzed isotopes ranging from ^7^Li to ^209^Bi. The report highlights calibration checkpoint parameters, baseline noise indices, and average recoveries for quality control evaluation. **(B)** Representative raw data report exported from the Qtegra software interface (thermo scientific iCAP RQ, serial no. RQ01815) for calibration standard 2 (“Std 2 MD”). The tables document the average elemental concentrations (ppb) and concentrations per individual for healthy pigeon pea leaves (ICPL 99090), analytical run across a broad suite of target isotopes spanning from ^7^Li to ^209^Bi to verify instrument stability and operational accuracy.

Supplementary Table 4 Comparative elemental concentration composition (μg/kg) in healthy (ICPL 99090) and infected (ICP8863) pigeon pea leaves as determined by ICP-MS.
