# Supplementary material for: Ionomic and metabolic signatures governing sterility mosaic disease resistance in pigeonpea
Source: Front Plant Sci. 2026 Sep 3;17:1870334. doi: 10.3389/fpls.2026.1870334 (PMC13582483; doi:10.3389/fpls.2026.1870334)
Supplement: Supplementary file 1 [file Supplementaryfile1.docx]

**SUPPLEMENTARY MATERIAL**

**Table 1 Seasonal weather parameters recorded during field trials.**

| **Year** | **Mean DI** | **T(max)** | **T(min)** | **RH (Morning)** | **RH (Evening)** | **Wind velocity** | **Sunshine hrs** | **Rainfall** |
| --- | --- | --- | --- | --- | --- | --- | --- | --- |
| 2022  **(SMW 25-31)** | 26.78 | 32.43261 | 22.01679 | 88.34883 | 60.03055 | 4.487323 | 6.229818 | 3.598788 |
| 2023  **(SMW 25-31)** | 24.3 | 33.45018 | 21.86576 | 86.73956 | 52.86751 | 4.626843 | 5.69703 | 1.017273 |

D.I- disease incidence; T (max)-maximum temperature; T (min)-minimum temperature; relative humidity;

SMW -Standard Meteorological week

**Fig. 1 ICPMS- Elemental calibration**

**Fig. 1A** Calibration curves for elemental analysis: Representative calibration curves obtained via ICP-MS for target isotopes ^107^Ag, ^111^Cd, ^115^In, ^137^Ba, ^205^Ti & ^208^Po in STD mode. Each panel displays the linear regression equation f(x), coefficient of determination (R^2^), background equivalent concentration (BEC) and limit of detection (LoD) across a concentration range from 0 to 250 ppb. Signal intensity is plotted as counts per second (cpsx10^4^).

**Fig. 1B**

**Fig. 1C**

**Fig. 1B & Fig. 1C** Representative calibration curves obtained via ICP-MS for target isotopes ^27^Al ^39^K,, ^209^Bi & ^44^Ca in STD mode. Each panel displays the linear regression equation f(x), coefficient of determination (R^2^), background equivalent concentration (BEC) and limit of detection (LoD) across a concentration range from 0 to 250 ppb. Signal intensity is plotted as counts per second (cpsx10^4^).

**Fig. 1D**

**Fig. 1E**

**Fig. 1D & Fig. 1E Representative** calibration curves obtained via ICP-MS for target isotopes ^23^Na ^24^Mg, ^55^Mn & ^57^Fe in STD mode. Each panel displays the linear regression equation f(x), coefficient of determination (R^2^), background equivalent concentration (BEC) and limit of detection (LoD) across a concentration range from 0 to 250 ppb. Signal intensity is plotted as counts per second (cpsx10^4^).

**Table 2A:	Screening of pigeonpea genotypes against sterility mosaic disease during *Kharif* 2022**

| **Sr No** | **Pedigree** | **Entry code** | **Symptoms** | **Per cent Disease Incidence** | **Disease Reaction** |
| --- | --- | --- | --- | --- | --- |
| 1 | ICPL 19018 | PP2201 | Mosaic | 32.0 | MS |
| 2 | ICPL 19074 | PP2202 | Mosaic | 15.0 | MR |
| 3 | ICPL 19063 | PP2203 | Leaf rolling | 12.0 | MR |
| 4 | ICPL 19042 | PP2204 | Chlorotic rings | 5.0 | R |
| 5 | ICPL 19054 | PP2205 | Chlorotic rings | 16.0 | MR |
| 6 | ICPL 19037 | PP2206 | Mosaic | 3.0 | R |
| 7 | ICPL 19047 | PP2207 | Mosaic | 12.0 | MR |
| 8 | ICPL 19076 | PP2208 | Leaf rolling, Chlorotic rings | 25.0 | MS |
| 9 | ICPL 19071 | PP2209 | Mosaic | 15.0 | MR |
| 10 | ICPL 19051 | PP2210 | Mottling | 12.0 | MR |
| 11 | ICPL 19069 | PP2211 | Mosaic | 16.0 | MR |
| 12 | ICPL 19039 | PP2212 | Mosaic | 18.0 | MR |
| 13 | ICPL 20096-1 | PP2213 | No symptoms | 5.0 | R |
| 14 | ICPL 20123 | PP2214 | Mosaic | 12.0 | MR |
| 15 | ICPL 20136 | PP2215 | Curling & Leaf rolling | 15.0 | MR |
| 16 | ICPL 99046 | PP2216 | Mosaic | 12.0 | MR |
| 17 | ICPL 99090 | PP2217 | No symptoms | 5.0 | R |
| 18 | ICPL 99092 | PP2218 | Curling & Leaf rolling, Mosaic | 15.0 | MR |
| 19 | ICPL 20114 | PP2219 | Mosaic, Chlorotic rings | 45.0 | S |
| 20 | ICPL 20124 | PP2220 | Mosaic | 30.0 | MS |
| 21 | ICPL 99048 | PP2221 | Chlorotic rings | 12.0 | MR |
| 22 | ICPL 99102 | PP2222 | Chlorotic rings | 50.0 | S |
| 23 | ICPL 2011229 | PP2223 | Mosaic | 30.0 | MS |
| 24 | ICP 2376 (Check) | ICPWSMD2232 | No symptoms | 3.0 | R |
| 25 | ICP 8863 (Check) | ICPWSMD2233 | Mosaic | 95.0 | S |

| **Fp** | **<0.001** |
| --- | --- |
| **LSD (P 0.05)** | **5.45** |
| **CV (%)** | **10.90** |

**Table 2B: Screening of Pigeon pea genotypes against sterility mosaic disease *Kharif* 2023**

| **Sr. No** | **Pedigree** | **Entry code** | **Symptoms** | **Per cent Disease**  **Incidence** | **Disease**  **Reaction** |
| --- | --- | --- | --- | --- | --- |
| 1. | ICPL 15038 | ICPWSMD2210 | Mottling | 15.0 | MR |
| 2. | ICPL 17005 | ICPWSMD2202 | Mosaic | 5 | R |
| 3. | ICPL 17017 | ICPWSMD2216 | No symptoms | 3.0 | R |
| 4. | ICPL 11229 | ICPWSMD2222 | Chlorotic rings | 5.0 | R |
| 5. | ICPL 99004 | ICPWSMD2204 | Chlorotic rings | 5.0 | R |
| 6. | ICPL 332 | ICPWSMD2203 | Leaf puckering | 15.0 | MR |
| 7. | ICPL 11227 | ICPWSMD2207 | Mosaic | 17.0 | MR |
| 8. | ICPL 11226 | ICPWSMD2206 | Mosaic | 16.0 | MR |
| 9. | ICPL 15018 | ICPWSMD2205 | No symptoms | 5.0 | R |
| 10. | ICPL 20096-1 | ICPWSMD2225 | No symptoms | 2.0 | R |
| 11. | ICP 2376 (Check) | ICPWSMD2232 | No symptoms | 5.0 | R |
| 12. | ICPL 99048 | ICPWSMD2212 | Mosaic, leaf puckering | 50.0 | MS |
| 13. | ICPL 15014 | ICPWSMD2209 | Mosaic | 5.0 | R |
| 14. | ICPL 99090 | ICPWSMD2228 | No symptoms | 2.0 | R |
| 15. | ICPL 20135 | ICPWSMD2215 | Mosaic | 10 | R |
| 16. | ICPL 2011228 | ICPWSMD2220 | Mosaic | 35.0 | MS |
| 17. | ICPL 15003 | ICPWSMD2208 | Leaf rolling, Chlorotic rings | 40.0 | MS |
| 18. | ICPL 15061 | ICPWSMD2213 | Mosaic | 35.0 | MS |
| 19. | ICPL 17004 | ICPWSMD2201 | Mosaic | 35.0 | MS |
| 20. | ICPL 2011220 | ICPWSMD2219 | Mosaic, Chlorotic rings | 50.0 | S |
| 21. | ICPL 15079 | ICPWSMD2214 | Mosaic | 18.0 | MR |
| 22. | ICPL 11236 | ICPWSMD2223 | Mosaic | 35.0 | MS |
| 23. | ICPL 11215 | ICPWSMD2221 | Chlorotic rings | 12.0 | MR |
| 24. | ICPL 17016 | ICPWSMD2217 | Leaf puckering | 12.0 | MR |
| 25. | ICPL 20124 | ICPWSMD2218 | Curling & Leaf rolling, Mosaic | 16.0 | MR |
| 26. | ICPL 17001 | ICPWSMD2224 | Mosaic | 18.0 | MR |
| 27. | ICPL 20123 | ICPWSMD2211 | Mosaic | 18.0 | MR |
| 28. | ICP 8863 (Check) | ICPWSMD2233 | Mosaic | 95.0 | S |
| 29. | ICPL 20114 | ICPWSMD2229 | Mosaic | 40.0 | MS |
| 30. | ICPL 94062 | ICPWSMD2227 | Chlorotic rings | 75.0 | S |
| 31. | ICPL 99102 | ICPWSMD2230 | Mosaic | 35.0 | MS |
| 32. | ICPL 84031 | ICPWSMD2231 | Chlorotic rings | 60.0 | S |
| 33. | ICPL 20125 | ICPWSMD2226 | Chlorotic rings | 18.0 | MR |

| **Fp** | **<0.001** |
| --- | --- |
| **LSD (P 0.05)** | **7.529** |
| **CV (%)** | **18.3** |

**Fig. (2A)**	**Fig. (2B)**

**Fig.2**	RT-PCR detection of PPSMV- 2 in infected pigeonpea leaf samples using RNA-2 (384 bp) and RNA-3 (284 bp) specific primers. **Fig. (2A)** Lane M: 100 bp DNA ladder; lanes 1-14 infected field samples; **Fig. (2B)** Lane M: 100-bp DNA ladder; lanes 1-5 infected field samples

**SEM- Biological Replicates**

**Fig.3A-B**

**Fig.3 C-D**

**Fig.3A-B & Fig.3 C-D**

SEM image representing the biological replicates of infected pigeon pea leaves used to assess surface structures, highlighting a dense distribution of non-glandular trichomes surrounding central particulate or cellular structures. The image confirms consistency in surface topography and structural integrity across independent biological replicates. Scale bar = 10 µm.

**Fig.3 E-F** SEM image representing a biological replicate of healthy **(E)** infected **(F)** pigeon pea leaves used to assess surface structures, highlighting a dense distribution of non-glandular trichomes surrounding central particulate or cellular structures. The image confirms consistency in surface topography and structural integrity across independent biological replicates. Scale bar = 10 µm.

**Fig.3 G-H**

**(G)** Scanning electron micrograph (1000x) capturing a detailed view of the surface micro-morphology of the pigeon pea leaves. The imaging field reveals an agglomeration of spherical micro-particles embedded within an amorphous matrix, demonstrating the local surface texture and structural arrangement of the sample. Scale bar = 10 µm.

**(H)** Low-magnification SEM imaging field of the pigeon pea leaf tissue. Wide-field scanning electron micrograph (45x) illustrating the macro-level surface topography and overall tissue architecture. This imaging field demonstrates the homogeneous distribution and dense coverage of micro-structures across a broad surface area of the biological specimen. Scale bar = 100 µm.

**Fig.3 I** Overview scanning electron micrograph (25x) displaying a broad macroscopic field of view across the pigeon pea leaf surface. The imaging field captures a major leaf vein running vertically alongside net-like minor venation networks, demonstrating the uniform distribution of surface microstructures and an isolated surface feature or deposit in the upper right quadrant.

Scale bar = 0.5 mm.

**FTIR spectra of 33 Genotypes.**

**Genotype -1	Genotype -2**

**Genotype -3	Genotype -4**

**Genotype -5	Genotype -6**

**Genotype -7	Genotype -8**

**Genotype -9	Genotype -10**

**Genotype -11	Genotype -12**

**Genotype -13	Genotype -14**

**Genotype -15	Genotype -16**

**Genotype -17	Genotype -18**

**Genotype -19	Genotype -20**

**Genotype -21	Genotype -22**

**Genotype -23	Genotype -24**

**Genotype -25	Genotype -26**

**Genotype -27	Genotype -28**

**Genotype -29	Genotype -30**

**Genotype -31	Genotype -32**

**Genotype -33**

**Fig. 4:** Fourier-transform infrared spectroscopy (FTIR) spectra of 33 genotypes evaluated during *Kharif* 2023 showing percentage transmittance across wavenumbers from 4000 to 400 cm⁻¹ along with key characteristic absorption bands including a broad peak around 3335 cm⁻¹ (O–H stretching), C–H stretching bands near 2917 cm⁻¹ and distinctive fingerprint region peaks (including 1731, 1636, and 1031 cm⁻¹ corresponding to the functional group.

**Table 3:**	FTIR band assignments and biochemical interpretations of spectral shifts in representative healthy (ICPL 99090) and PPSMV-2 infected (ICP8863) pigeonpea leaves.

| **Wave**  **number (cm⁻¹)** | **Band assignment** | **Healthy samples** | **Infected samples** | **Biochemical interpretation** |
| --- | --- | --- | --- | --- |
| ~3330 | O-H / N-H stretching (hydrogen bonding, phenolics, proteins) | Relatively narrow band with moderate intensity | Broadened band with relatively increased intensity | Increased hydrogen bonding and possible accumulation of phenolic/lignin-associated compounds under infection stress |
| ~2925 | C-H stretching (lipids, carbohydrates) | Well-defined band | Reduced band intensity | Altered lipid-associated metabolism and possible membrane-associated changes |
| ~1640 | Amide I (C=O stretching of proteins) | Distinct band | Reduced intensity with slight band shift | Alterations in protein structure and conformation under infection stress |
| ~1540 | Amide II (N-H bending and C-N stretching of proteins) | Clearly detectable band | Reduced or less distinct band | Changes in structural and defense-associated proteins |
| ~1410 | C-H bending / phenolic O-H | Lower band intensity | Relatively increased band intensity | Possible accumulation of phenolic compounds |
| ~1050 | C-O-C stretching (polysaccharides, cell wall components) | Moderate band intensity | Relatively enhanced band intensity | Altered carbohydrate metabolism and possible cell wall remodeling |

**Fig. 5A:** ICP-MS raw concentration and intensity data report of infected pigeon pea leaves (ICP 8863) generated via Qtegra software (Thermo Scientific iCAP RQ) displaying elemental concentrations (ppb), average signal intensities (counts per second, cps) and relative standard deviations (RSD) for analyzed isotopes ranging from ^7^Li to ^209^Bi. The report highlights calibration checkpoint parameters, baseline noise indices, and average recoveries for quality control evaluation.

**Fig. 5B:** Representative raw data report exported from the Qtegra software interface (Thermo Scientific iCAP RQ, Serial No. RQ01815) for calibration standard 2 ("Std 2 MD"). The tables document the average elemental concentrations (ppb) and concentrations per individual for healthy pigeon pea leaves (ICPL 99090), analytical run across a broad suite of target isotopes spanning from ⁷Li to ²⁰⁹Bi to verify instrument stability and operational accuracy.

Table 4	Comparative elemental concentration composition (μg/kg) in healthy (ICPL 99090) and infected (ICP8863) pigeon pea leaves as determined by ICP-MS.

| **Element** | **Healthy**  (μg/kg) | **Infected**  (μg/kg) | **Fold Change** | **Detection Limit**  parts per trillion (ppt) |
| --- | --- | --- | --- | --- |
| Na | 6.85 | 5.31 | 1.29 | 28 |
| Mg | 6.17 | 2.77 | 2.22 | 0.7 |
| Al | 4.98 | 2.74 | 1.81 | 3.3 |
| K | 10.11 | 8.32 | 1.21 | 10 |
| Ca | 7.83 | 1.84 | 4.25 | 20 |
| Fe | 3.01 | 1.21 | 2.48 | 349 |
| Mn | 3.37 | 0.38 | 8.86 | 0.7 |
| Zn | 1.79 | **< LOD** |  | 2.9 |
| Ni | **< LOD** | 0.47 |  | 2.0 |
| Cd | **< LOD** | 0.60 |  | 0.2 |
